# Microenvironment Engineering Enables Broad Strategies for Lithium‐Sulfur Batteries

**DOI:** 10.1002/advs.202505685

**Published:** 2025-07-11

**Authors:** Ranqi Li, Yingbo Xiao, Shaoming Huang

**Affiliations:** ^1^ School of Chemistry and Materials Science Hangzhou Institute for Advanced Study University of Chinese Academy of Sciences Hangzhou 310024 China; ^2^ Zhejiang Key Laboratory of Alternative Technologies for Fine Chemicals Process School of Chemistry and Chemical Engineering Shaoxing University Shaoxing 312000 China

**Keywords:** lithium‐sulfur batteries, microenvironment engineering, structural, lithiophilicity, sulfiphilicity

## Abstract

Lithium‐sulfur batteries (LSBs) are regarded as one of the most promising next‐generation energy storage technologies due to their high energy density, abundant resource availability, and environmental sustainability. However, significant challenges, such as the lithium polysulfides shuttle effect and sluggish redox kinetics, impede their practical application. Microenvironment engineering provides innovative solutions to these issues by precisely controlling the physical and chemical environments of key components within LSBs. Despite the significance of this approach, there is a lack of systematic reviews on its application in the field of LSBs. This review fills the gap by comprehensively summarizing the research progress in microenvironment engineering for LSBs, focusing on four key aspects: 1) structural microenvironment engineering; 2) lithiophilicity microenvironment engineering; 3) sulfiphilicity microenvironment engineering; and 4) lithiophilicity‐sulfiphilicity microenvironment engineering. These strategies are analyzed for their role in mitigating the challenges associated with LSBs. Finally, the research directions and the ongoing potential of the microenvironment engineering to drive further progress in this field are proposed for inspiring innovation and accelerating the practical application of LSBs in future.

## Graphical abstract

Microenvironment engineering offers effective solutions to the challenges of lithium‐sulfur batteries (LSBs) by controlling the physical and chemical environments of their key components. This review highlights the progress in four microenvironment engineering strategies: structural, lithiophilicity, sulfiphilicity, and lithiophilicity‐sulfiphilicity microenvironment engineering. It examines the role of these strategies in addressing the challenges of LSBs and proposes potential directions for future research.

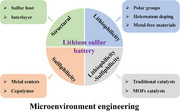

## 1 Introduction

With the advancement of society and the rapid development of science and technology, there is an urgent need for advanced energy storage devices to meet the growing demand in emerging fields.^[^ ^1^ ^]^ Among the available options, lithium‐ion batteries (LIBs) have gained widespread use in portable electronics, electric vehicles, aerospace, and other fields due to their low self‐discharge rates and long cycle life.^[^ ^2^, ^3^, ^4^, ^5^ ^]^ However, current LIBs face limitations in high‐energy storage applications, primarily due to the high cost of cathode materials and relatively low energy density.^[^ ^6^, ^7^ ^]^ Therefore, it is essential to develop energy storage devices with high energy density and low cost to meet the demands of industrial development. In this context, lithium‐sulfur batteries (LSBs), which use sulfur as the cathode and lithium metal as the anode and offer a theoretical specific capacity of 1675 mAh g^−1^ and a theoretical specific energy of 2600 Wh kg^−1^, have gained significant attention.^[^ ^8^, ^9^, ^10^ ^]^ Additionally, sulfur is abundant, inexpensive, and non‐toxic, making LSBs a promising alternative for high‐energy storage applications and a potential candidate for next‐generation secondary batteries.^[^ ^11^, ^12^ ^]^

Despite these advantages, the commercialization of LSBs remains hindered by several critical challenges: 1) unstable electrode structure; 2) severe shuttle effect; 3) sluggish redox kinetics; 4) limited practical energy density. To address these issues, extensive research has focused on the rational design of the microenvironment of key components within LSBs. For instance, introducing non‐polar carbon materials (carbon nanotubes (CNTs),^[^ ^13^, ^14^, ^15^ ^]^ graphene,^[^ ^16^, ^17^ ^]^ porous carbon^[^ ^18^, ^19^, ^20^, ^21^, ^22^ ^]^) to structurally optimize sulfur hosts, thus buffering volume changes and maintaining electrode stability while enabling high sulfur loading. Additionally, incorporating conductive interlayers between the cathode and separator can promote ion and electron transport, physically confining lithium polysulfides (LiPSs) near the cathode and preventing their migration to the anode.^[^ ^23^ ^]^ Based on these strategies, some of the challenges have been addressed and the overall performance of LSBs has been significantly improved to some extent. Nevertheless, it is worth noting that due to the weak interaction of LiPSs within the batteries, particularly on the cathode side, the shuttle effect still exists and inevitably contributes to the low sulfur utilization and the poor cycling stability of LSBs.^[^ ^24^ ^]^

To further suppress the shuttle of LiPSs and enhance the overall performance of LSBs, it is essential to incorporate the concept of chemical affinity into the microenvironmental engineering of LSBs.^[^ ^25^ ^]^ In terms of lithium affinity, a lithophilic microenvironment can be established by introducing pristine metal‐free materials, doping non‐polar heteroatoms, or modifying polar functional groups in various components, such as sulfur hosts, interlayers, and separators. These approaches not only mitigate LiPSs shuttling but also accelerate lithium‐ion transfer and extend battery cycle life. Regarding sulfur affinity, a sulfiphilicity microenvironment can be constructed by leveraging materials such as different forms of metal oxides,^[^ ^26^ ^]^ sulfides,^[^ ^27^ ^]^ nitrides,^[^ ^28^ ^]^ carbides,^[^ ^29^ ^]^ phosphides,^[^ ^30^ ^]^ and metal‐organic frameworks.^[^ ^31^ ^]^ These materials facilitate the anchoring and catalytic conversion of LiPSs, effectively suppressing the shuttling effect. However, relying solely on either lithium or sulfur affinity is insufficient to simultaneously optimize charge/discharge efficiency and minimize the shuttle effect. Therefore, the rational design of dual‐function LSBs that combine both lithophilicity and sulfiphilicity is of great significance. According to recent research, several promising strategies for achieving dual chemical affinity are overviewed as follows: 1) traditional catalysts structure regulation via heterostructure construction, multicomponent incorporation, vacancy, doping, and coordination engineering; 2) Metal‐organic frameworks (MOFs) catalysts structure regulation by defect engineering, confinement effect, and integrated design. These approaches enable the simultaneous optimization of lithium and sulfur interactions, paving the way for more efficient and durable LSBs.

**Figure 1:**
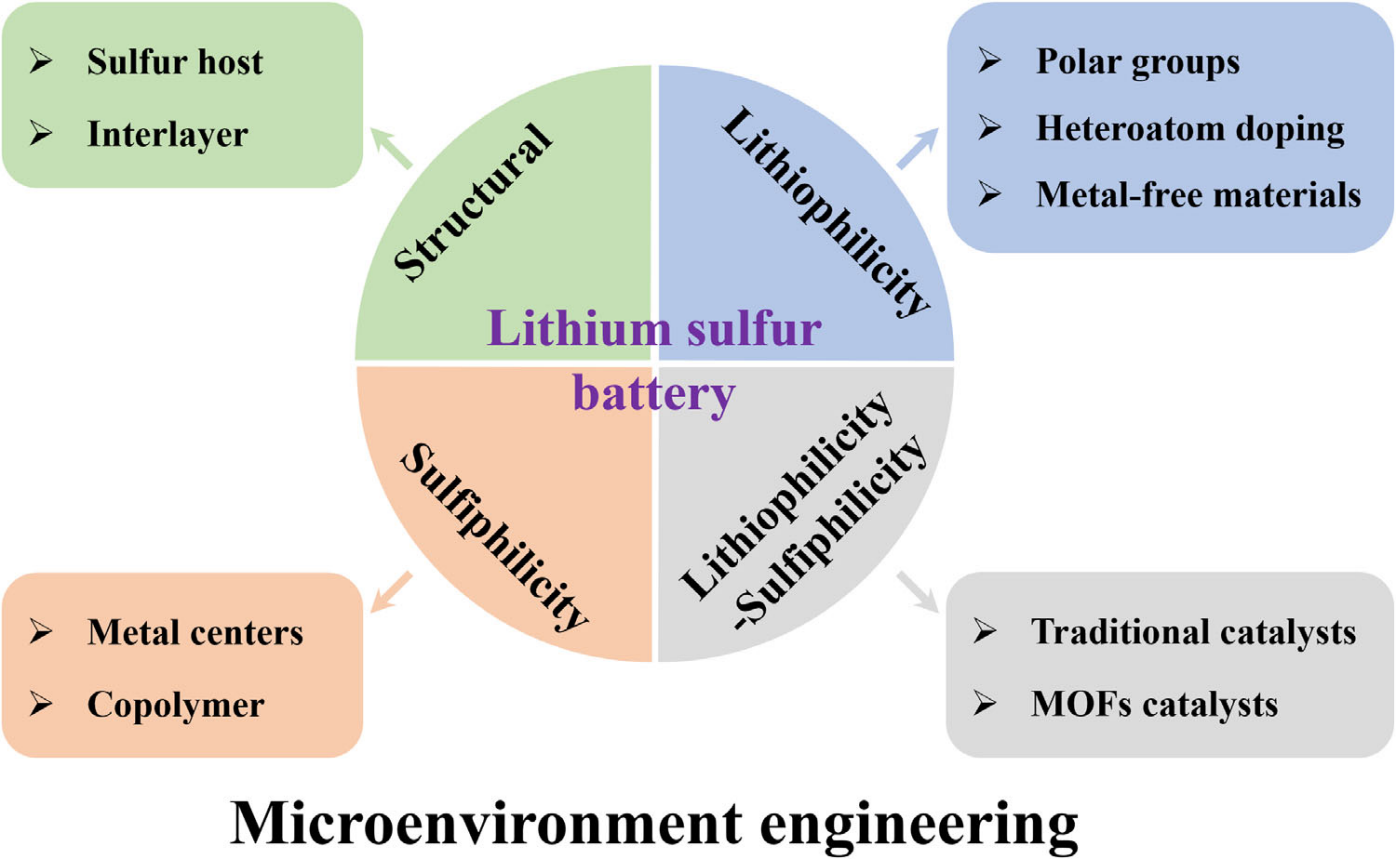
Microenvironment engineering for LSBs: structural microenvironment engineering, lithiophilicity microenvironment engineering, sulfiphilicity microenvironment engineering, and lithiophilicity‐sulfiphilicity microenvironment engineering.

In recent years, microenvironmental engineering has garnered significant attention and provided broad strategies for addressing the challenges associated with LSBs. Although a few reviews have explored specific aspects such as structural optimization or functional site design in LSBs,^[^ ^32^, ^33^, ^34^ ^]^ however, comprehensive and systematic summary of microenvironmental engineering in LSBs remains scarce. Herein, this review seeks to fill this gap by providing a detailed overview of recent advancements in microenvironmental engineering for LSBs, highlighting its related various strategies to enhance the understanding of LSBs. The review begins with an introduction to the fundamental principles and challenges of LSBs, which sets the stage for understanding the role of microenvironment engineering. Subsequently, the microenvironmental engineering of LSBs is discussed in detail from four perspectives: 1) Structural microenvironment engineering; 2) Lithiophilicity microenvironment engineering; 3) Sulfiphilicity microenvironment engineering; 4) Lithiophilicity‐sulfiphilicity microenvironment engineering. Finally, the outlooks and potential directions for LSB development and further research are proposed (**Figure** **1**).

## 2 Fundamental Principles and Challenges of Lithium‐Sulfur Batteries

### 2.1 Fundamental Principles

**Figure 2:**
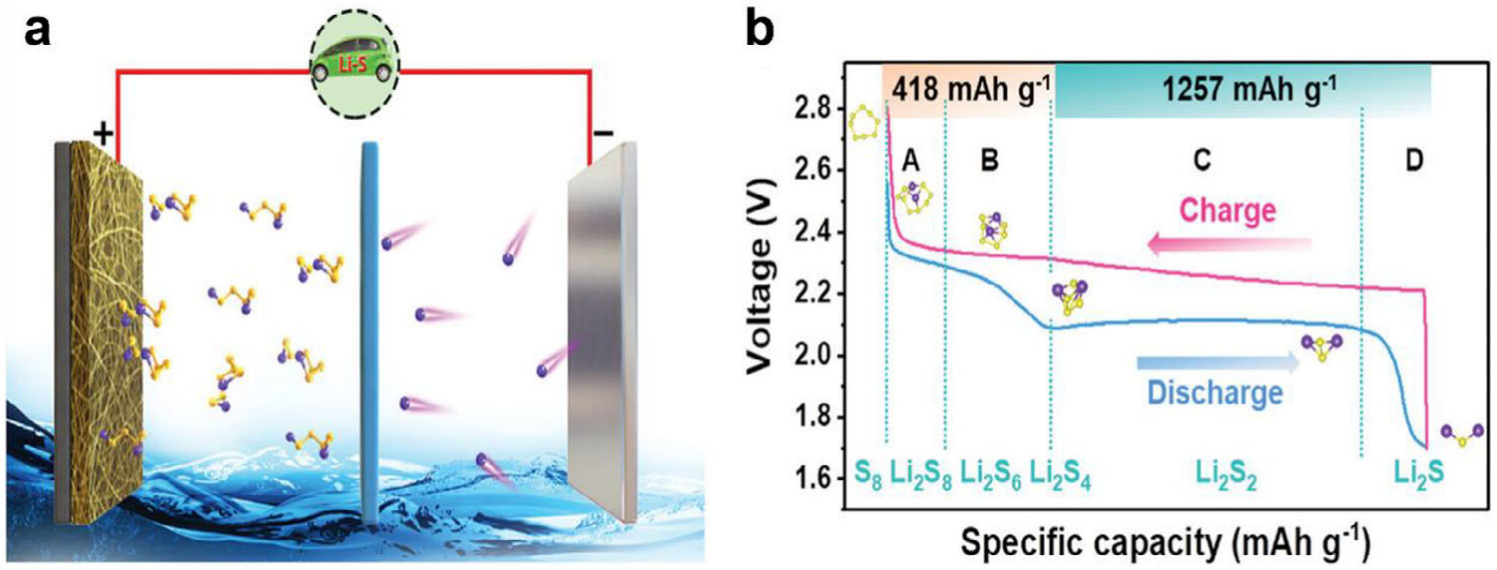
a) Schematic illustration of LSBs. b) The discharge/charge curves of LSBs. (a,b) Reproduced with permission.^[^ ^25^ ^]^ Copyright 2023, Wiley.

The operation of LSBs relies on the redox reaction between sulfur (S_8_) and lithium metal to achieve energy storage and release (**Figure** **2**a). This mechanism differs from the intercalation‐deintercalation electrochemical reaction that occurs in LIBs.^[^ ^35^ ^]^ The corresponding reaction equations for both the electrodes and the battery are as follows:

(1) $$\mathrm{Cathode}:\;S_{8}+16Li^{+}+16e^{-}↔8Li_{2}S$$

(2) $$\mathrm{Anode}:\;\mathrm{Li}↔Li^{+}+e^{-}$$

(3) $$\mathrm{Overall}\;\mathrm{reaction}:\;S_{8}+16\mathrm{Li}↔8Li_{2}S$$

Typically, the discharge process of LSBs is inherently more complex than the charging process, encompassing two distinct reaction stages. As shown in Figure 2b, the discharge curve consists of a higher voltage plateau (2.1–2.4 V), where S_8_ undergoes ring‐opening reaction and is lithiated, forming soluble long‐chain LiPSs (Li_2_S_8_, Li_2_S_6_, Li_2_S_4_). This stage contributes ≈25% of the theoretical capacity (418 mAh g^−1^). Subsequently, these LiPSs are reduced to insoluble short‐chain polysulfides (Li_2_S_2_ and Li_2_S), which corresponds to the lower voltage plateau (≈2.1 V) and contributes ≈75% of the theoretical capacity (1257 mAh g^−1^).

During charging, the reverse reactions occur: Li_2_S is oxidized back to S_8_ through intermediate LiPSs, while lithium ions (Li‐ion) are deposited at the anode upon accepting electrons, enabling the reversible discharge/charge process. Notably, this reversible discharge/charge cycle involves the transfer of 16 electrons and proceeds through a complex “solid‐liquid‐solid” conversion, distinguishing LSBs from other battery systems.^[^ ^9^, ^36^ ^]^

### 2.2 Challenges

LSBs also face a series of challenges. These issues are summarized below:

1. Unstable electrode structure. Due to the density difference between S_8_ and Li_2_S, active materials suffer ≈80% volume expansion during the lithiation process.^[^ ^37^ ^]^ More importantly, the cathode volume dramatically changes, continually expanding and contracting during the long‐term discharge/charge process, which will seriously damage the original structure of electrode, leading to the pulverization of the electrode and the low cycle life of batteries.
2. Severe shuttle effect. Generally, the sulfur reaction involves two stages. The intermediate LiPSs (Li_2_S_8_, Li_2_S_6_, Li_2_S_4_) formed during the first stage are easily dissolved in the electrolyte, and subsequently migrate back and forth between the cathode and the anode owing to the presence of the electric field and concentration gradient.^[^ ^38^ ^]^ The above process is the inevitable shuttle effect in LSBs, which is the main reason for low sulfur utilization, poor Coulombic efficiency (CE), terrible cycling performance, and rapid capacity decay.
3. Sluggish redox kinetics. The low conductivity of sulfur (5 × 10^−10^ S cm^−1^) and the discharge product Li_2_S (1 × 10^−13^ S cm^−1^) severely affects the transport of electrons and Li‐ion, leading to sluggish redox kinetics.^[^ ^39^, ^40^, ^41^ ^]^ In addition, due to the shuttle effect, the long‐chain LiPSs migrate from the cathode to the anode and react with lithium metal to deposit on the anode surface, which not only results in the loss of active materials but also inhibits the anode reaction rate and further decelerates the redox kinetics.^[^ ^42^, ^43^, ^44^ ^]^
4. Limited practical energy density. Although LSBs have a theoretical specific energy of 2600 Wh kg^−1^, their practical energy density is severely limited and cannot even reach the level of commercial LIBs.^[^ ^45^ ^]^ On the one hand, the above three problems, especially the shuttle of LiPSs, significantly reduce the utilization of active materials and decrease the amount of sulfur, thereby leading to the actual energy density of LSBs far below the theoretical value. On the other hand, most LSBs are evaluated under testing conditions such as low electrolyte/sulfur (E/S) ratio. At this time, the sulfur loading of the cathode is generally less than 2 mg cm^−2^, which makes it hard to realize the requirement of high energy density.^[^ ^34^ ^]^

Overall, these problems seriously affect their electrochemical performance and hinder the commercialization of LSBs. Therefore, it is important that develop microenvironmental engineering of LSBs, which is the key to overcoming the above issues and improving the electrochemical performance. Various microenvironmental engineering and its related strategies will be discussed in detail in the following sections.

## 3 Structural Microenvironment Engineering

Structural microenvironmental engineering is a straightforward and effective protocol for improving the overall performance of LSBs. Recently, most studies have focused on the structural optimization of sulfur hosts and interlayers, which will be introduced in the following sections, respectively.

### 3.1 Structural Optimization of Sulfur Hosts

Sulfur hosts play an important role in LSBs since they can provide enough space to accommodate large amounts of sulfur and volume change of the cathode, and physically confine the dissolution and diffusion of LiPSs. In addition, they can also improve the conductivity of active materials, accelerating electron and Li‐ion transport, thus promoting the redox kinetics of LSBs. As a result, considerable efforts have been dedicated to the methodical design of sulfur hosts, with a focus on their structural aspects (**Table** **1**).^[^ ^20^, ^46^, ^47^, ^48^, ^49^, ^50^, ^51^, ^52^, ^53^, ^54^ ^]^

**Table 1:** Summary of structural optimization of sulfur hosts in LSBs.

Material	Sulfur content [wt%]	Sulfur loading [mg cm^−2^]	Rate [C]	Cycle number	First discharge capacity [mAh g^−1^]	Discharge capacity after cycling [mAh g^−1^]	Refs.
FCNS/S	81	N/A	1	200	1104	922.5	[20]
ABPC‐w/S	75	N/A	0.1	100	1498	1191	[46]
CC@S70	70	1	0.3	260	1405	798	[47]
GPC/HT‐S	87.6	N/A	0.5	400	820	592	[48]
CNTF/S	N/A	0.9	0.2	1000	1344	713	[49]
CNT/graphene/S	64.9	2.18	0.5	1500	1151.2	425.9	[50]
2850CNTs‐Gra‐S	65.5	N/A	15	1500	314	273	[51]
S/MOF‐76(Gd) (C)	N/A	2.1	0.5	200	657.9	610.2	[52]
Py‐COF/S	70	0.8‐1.2	5	550	652	481.2	[53]
CAGE‐COF/S	70	1.2‐1.5	1	500	728	573	[54]

#### 3.1.1 Nonpolar Carbon Materials as Sulfur Hosts

**Figure 3:**
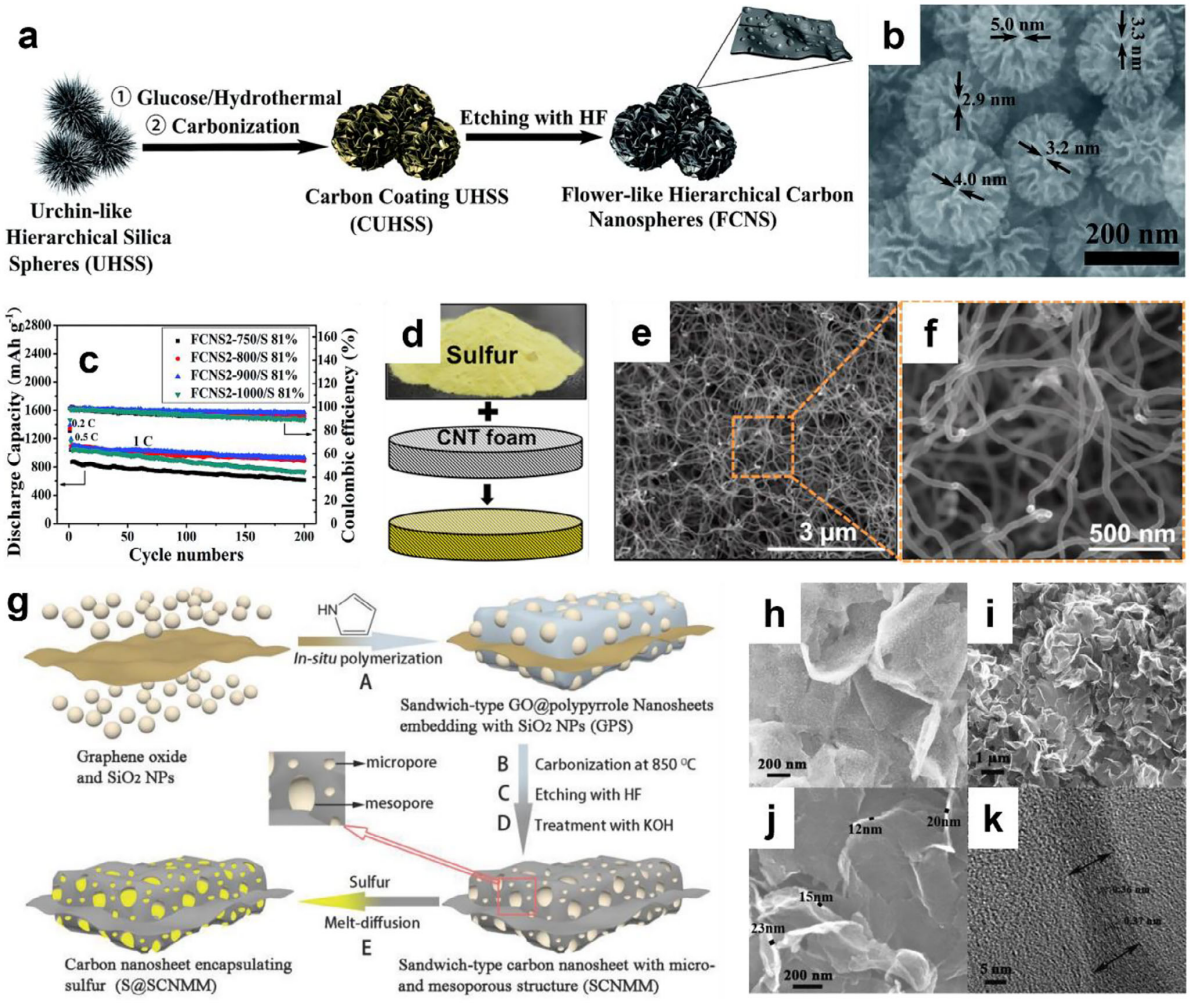
a) Schematic illustration of the procedure for preparing FCNS. b) scanning electron microscope (SEM) images of FCNS2‐900. c) Cycle performance of S/FCNS at 1 C. (a–c) Reproduced with permission.^[^ ^20^ ^]^ Copyright 2017, Royal Society of Chemistry. d) Schematic drawing of sulfur impregnation on the CNTF. e) SEM image of the interconnected CNT network. f) Magnification of the orange squared region in e). (d–f) Reproduced with permission.^[^ ^49^ ^]^ Copyright 2018, Elsevier. g) Schematic illustration of the procedure for preparing S@SCNMM. h) SEM image of SiO_2_ NPs inserted‐hybrid carbon nanosheet. i,j) SEM images at different magnification of SCNMM. k) Cross sectional HRTEM image of SCNMM. (g,k) Reproduced with permission.^[^ ^19^ ^]^ Copyright 2014, Wiley.

Owing to their remarkable electrical conductivity, substantial specific surface area, and strong chemical stability, carbon materials are commonly employed as hosts for sulfur to optimize the cathode structure of LSBs. Among various carbon‐based materials, porous carbon materials with high porosity and extensive specific surface areas have been widely investigated. This unique structure not only maximizes the storage of active materials to ensure high sulfur content but also provides rapid transport channels for Li‐ion and electrons. More importantly, the nanometer‐scale pores can suppress the diffusion of LiPSs and mitigate the capacity loss caused by the shuttle effect, ultimately improving the cycling stability of LSBs. Huang's group developed flower‐like hierarchical carbon nanospheres (FCNS) as sulfur hosts via a controllable synthesis approach (**Figure** **3**a).^[^ ^20^ ^]^ This unique petal‐like structure formed numerous mesoporous channels to promote the penetration of the electrolyte, which was favorable for the rapid migration of Li‐ion and electrons (Figure 3b). In addition, FCNS with a large specific surface area (1151 m^2^ g^−1^) and high pore volume (1.95 cm^3^ g^−1^) realized the physical confinement of LiPSs, reducing the diffusion of LiPSs in the electrolyte. Aa a result, the FCNS/S composite cathode exhibited a capacity retention of 922.5 mAh g^−1^ after 200 cycles at 1 C (Figure 3c).

Carbon nanotubes (CNTs) have been regarded as promising sulfur hosts because they can form conductive networks to facilitate the rapid transfer of ions and electrons. In addition, their extraordinary mechanical properties enhance the structural stability of the cathode and alleviate the volume expansion generated during discharge, thereby preventing electrode collapse and battery failure. Ummethala et al. prepared a free‐standing 3D carbon nanotube foam (CNTF) by a single‐step spray pyrolysis technique (Figure 3d).^[^ ^49^ ^]^ As shown in Figure 3e,f, the interconnected CNT network offered highly conductive channels for transporting electrons and facilitating redox kinetics. More importantly, the 3D structure of CNTF also effectively captured LiPSs dissolved in the electrolyte and physically confined LiPSs near the cathode, thus suppressing the severe shuttle effect and achieving higher sulfur utilization.

To further optimize the structure of sulfur hosts, hybrids of various carbon materials can be employed as a substitute for single carbon materials. Zhou et al. successfully reported the CNT/graphene composite cathode by growing CNTs on the graphene substrate.^[^ ^50^ ^]^ The graphene provided efficient and unobstructed pathways to transport ions and electrons. Additionally, the tubular CNTs effectively repaired the defects in the graphene and offered enough space to load more sulfur while accommodating volume changes. Huang's group demonstrated the fabrication of thin hybrid carbon nanosheets that are rich in micropores and mesopores (Figure 3g–k).^[^ ^19^ ^]^ The incorporation of nanosized micropores and mesopores into these thin nanosheets enhances the accessibility for the swift diffusion of organic electrolyte ions, facilitates efficient sulfur penetration, and reduces the dissolution and shuttle of LiPSs within the electrolyte. This integrated design of the carbon material produces synergistic effects, resulting in a high capacity and excellent cycling stability.

#### 3.1.2 Porous Materials as Sulfur Hosts

Porous materials, including MOFs and covalent‐organic frameworks (COFs), have strong potential as sulfur hosts in structure optimization due to their highly tunable porous structures and excellent specific surface areas. Capkova et al. synthesized MOF‐76(Gd) with a microporous structure and subsequently carbonized at 700 °C to act as a sulfur host in LSBs.^[^ ^52^ ^]^ The MOF‐76(Gd) derivative exhibited a higher specific surface area and porosity, which benefited the uniform loading of sulfur and the penetration of electrolytes. The retained flexible pore channels could accommodate the volume changes of sulfur, thereby maintaining the mechanical integrity of the cathode structure. More importantly, the synergistic effect of conductive carbonaceous and porous structures could improve the ionic transport capabilities of the electrode while effectively capturing LiPSs to mitigate the severe shuttle effect. Due to the above attributes, the cathode composed of carbonized MOF‐76(Gd) exhibited an impressive discharge capacity and retained a high‐capacity retention. These results provided novel strategies for the development of advanced cathodes in LSBs, especially for high‐energy‐density applications.

Apart from the MOFs, Covalent Organic Frameworks (COFs) constructed from organic monomers linked by covalent bonds also represent promising candidate hosts for sulfur attributed to their exceptional porosity, low density, and remarkable chemical stability. The extensive porosity can accommodate more sulfur to achieve high energy density and effectively trap dissolved LiPSs to limit the shuttle behavior. Meanwhile, the robust structure can buffer volume expansion, ensure the long‐term stability of the electrode. Recently, Meng et al. constructed a pyrene‐based 2D COF (Py‐COF) as a sulfur host in LSBs by the melt‐diffusion method, achieving up to 70 wt.% sulfur content.^[^ ^53^ ^]^ Such a remarkable sulfur loading can be attributed to the high specific surface area (2093 m^2^ g^−1^) and substantial pore volume (1.25 cm^3^ g^−1^) of the Py‐COF, which facilitated the uniform distribution of sulfur within the nanopores of the framework. Furthermore, the ordered porous structure of Py‐COF provided an ideal pathway for the efficient diffusion of Li‐ion, significantly improving the reaction kinetics of the battery. As a result, the resulting cathode exhibited excellent high‐rate performance and cycling stability, maintaining a high reversible capacity of 481.2 mAh g^−1^ even after 550 cycles at 5 C. This performance was notably superior to that of the commercial porous carbon BP2000/S electrode (only 265 mAh g^−1^ after 220 cycles). Unfortunately, 2D COFs have limited ability to confine LiPSs and cannot suppress the shuttle effect well. Therefore, 3D COFs are more attractive as sulfur hosts because they possess more complex and diverse pores, which can better physically restrict LiPSs and enhance sulfur utilization, thereby improving the electrochemical performance of LSBs. Wang et al. developed a 3D COF named CAGE‐COF by crosslinking of 2D COF (ART‐COF).^[^ ^54^ ^]^ In contrast to the original 2D COF, CAGE‐COF exhibited a larger specific surface area (298.27 m^2^ g^−1^), more open pore volume (0.185 cm^3^ g^−1^), and hierarchical pore sizes (1.26/1.77 nm), which collectively enabled uniform sulfur distribution and high sulfur loading capacity. Moreover, the unique cage‐like structure effectively encapsulated sulfur species and enhanced the physical confinement of LiPSs, dramatically restricting the shuttle effect.

In summary, conductive carbon materials and porous MOFs/COFs achieve great success as sulfur hosts, which is crucial for constructing the structural microenvironment. Notably, while these sulfur hosts increase sulfur loading, they also result in an increased mass of inactive components, leading to a reduction in sulfur content, adversely affecting the energy density of LSBs. Therefore, it is necessary to consider both the sulfur hosts and the active materials content to maximize the electrochemical performance of LSBs in practical applications.

### 3.2 Structural Optimization of Interlayers

Besides the above strategies, the structural optimization of interlayers is an alternative viable approach to developing structural microenvironmental engineering. Typically, the interlayer between cathode and separator acts as a physical barrier, restricting LiPSs to the cathode side, thus inhibiting the inevitable shuttle effect in LSBs. Furthermore, the high‐conductivity design of interlayers can enhance the transport rate of Li‐ion and significantly improve electrochemical reaction kinetics.

#### 3.2.1 Carbon‐Based Interlayers

**Figure 4:**
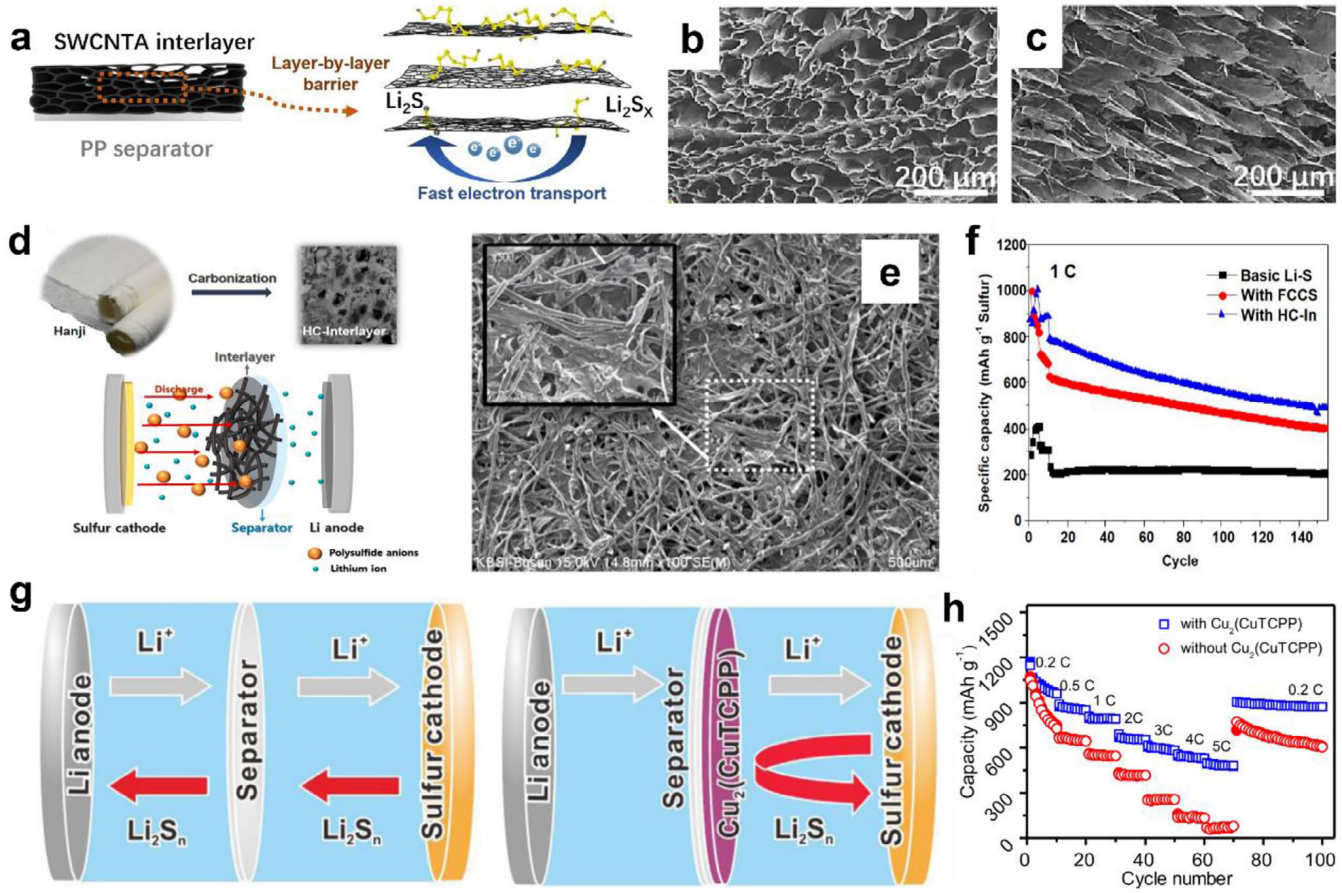
a) Schematic illustration of the physical blocking mechanisms of SWCNTA interlayers. SEM images of hierarchical pore structures in SWCNTAs b) radial direction, c) vertical direction). (a–c) Reproduced with permission.^[^ ^55^ ^]^ Copyright 2023, Elsevier. d) Schematic of LSBs with HC interlayer. e) SEM images of HC‐In. Cycling performance f) at 0.5 C. (d–f) Reproduced with permission.^[^ ^56^ ^]^ Copyright 2024, IOP Publishing. g) Schematic illustration of Cu_2_(CuTCPP) cells. h) Rate performance of the Cu_2_(CuTCPP) cells with the CB/S cathodes. (g,h) Reproduced with permission.^[^ ^58^ ^]^ Copyright 2019, Elsevier.

Traditional carbon materials, such as graphene, carbon nanotubes, and carbon nanofibers, have been widely employed in the structural optimization of interlayers. They provide conductive pathways for ion diffusion and promote the electrochemical reaction kinetics. Additionally, their unique nanostructure can intercept dissolved LiPSs, thereby indirectly stabilizing the lithium anode and improving the cycling performance of LSBs. Li et al. prepared single‐walled carbon nanotube aerogels (SWCNTAs) via freeze casting and applied them as interlayers in LSBs.^[^ ^55^ ^]^ The hierarchical lamellar structure of SWCNTAs provided a layer‐by‐layer physical barrier to confine polysulfide shuttling and protect the lithium anode (**Figure** **4**a), while their super wettability enhanced electrolyte infiltration and accelerated lithium‐ion transport (Figure 4b,c). Accordingly, the LSBs with the SWCNTA interlayers demonstrated excellent cycling performance and capacity retention, which brought rational inspiration for optimizing interlayer structures in LSBs.

Recently, carbon materials derived from biomass have gained significant attention as promising interlayers, owing to their environmental friendliness, low cost, and simple preparation process. Choi et al. developed a porous carbon interlayer (HC‐In) by carbonizing “hanji” to enhance the electrochemical performance of LSBs (Figure 4d).^[^ ^56^ ^]^ The HC‐In, characterized by its porous network structure, served as an effective polysulfide‐trapping barrier, preventing the migration of soluble LiPSs toward the anode (Figure 4e). Meanwhile, the conductive carbon fibers within the HC‐In could reduce the interfacial reaction resistance and accelerate the movement of electrons/ions, thus achieving rapid redox kinetics. Consequently, the LSBs with such interlayer exhibited high initial capacities of 873.11 mAh g^−1^ at 1 C (Figure 4f). Given the intact and continuous fiber network in waste fruit peels, the carbon‐based interlayers derived from these peels also show great potential in inhibiting the migration of LiPSs. Hu et al. proposed a free‐standing porous carbon film derived from waste pitaya peel (PPC film) as a functional interlayer for LSBs.^[^ ^57^ ^]^ The PPC film exhibited a hierarchical micro‐mesoporous architecture, which not only facilitated electrolyte infiltration and ion transport but also confined LiPSs diffusion through physical trapping. Furthermore, the abundant oxygen‐containing functional groups could further adsorb LiPSs and reduce the loss of active materials. Therefore, the cathode with PPC film interlayer attained a high sulfur loading of 3.5 mg cm^−2^ and showed capacity retention of 53.9% after 300 cycles at 0.5 C.

#### 3.2.2 MOF and COF‐Derived Interlayers

MOFs play a crucial role in the rational design of interlayers due to their highly ordered frameworks, appropriate pore size, and tunable pore parameters. Particularly, the selective sieving properties can effectively intercept LiPSs while ensuring the rapid transport of Li‐ion. Therefore, MOF‐derived interlayers can serve as physical barriers to regulate the migration of LiPSs and Li‐ion, thereby suppressing the shuttle effect. Tian et al. developed a highly oriented microporous membrane assembled from ultrathin Cu_2_(CuTCPP) MOF nanosheets via surfactant‐free wet‐chemistry synthesis and subsequent vacuum filtration (Figure 4g).^[^ ^58^ ^]^ The MOF membrane, with its controlled structural parameters and favorable mechanical flexibility, served as a sieve to concentrate LiPSs on the cathode side. The hierarchical architecture, characterized by aligned nanosheets stacked in a “brick wall” configuration, effectively captured LiPSs while ensuring rapid Li‐ion transport. When evaluated as an interlayer in the CB/S cathode, the copper‐based MOF membrane exhibited high‐rate capability (Figure 4h).

COFs are also considered favorable candidates for interlayers due to their highly tunable pore structure along with the ability to block polysulfide shuttling. Ma et al. proposed a cationic covalent organic framework nanosheet (CON‐TFSI) as an interlayer coated on a separator for LSBs.^[^ ^59^ ^]^ The CON‐TFSI layer possessed an ideal physical sieving mechanism, resulting from its ultrasmall pore size. Such a structure effectively blocked dissolved LiPSs from passing through the separator without affecting Li‐ion migration. The CON‐TFSI/PP batteries displayed an initial discharge capacity of 891.9 mAh g^−1^ at 0.2 C and maintained a specific capacity of 487.4 mAh g^−1^ (54.6%) after 500 cycles, significantly outperforming the unmodified PP batteries (4.3%) under identical test conditions.

Recently, the structure of interlayers has been rationally optimized with the aid of carbon materials or MOFs/COFs, and much progress has been made (**Table** **2**).^[^ ^55^, ^56^, ^57^, ^58^, ^59^, ^60^, ^61^, ^62^, ^63^, ^64^, ^65^ ^]^ Notably, there are still some obstacles to these strategies. On the one hand, although conventional carbon‐based interlayers possess good conductivity, they confine LiPSs poorly and cannot alleviate the shuttle effect well due to their low porosity and relatively flat structure. On the other hand, MOF/COF‐derived interlayers can effectively capture LiPSs, impeding the migration of LiPSs. However, their poor electrical conductivity may hinder ion transport. Considering the above issues, more attention should be paid to the synergistic design of conductive carbon materials and MOFs/COFs to optimize the structure of interlayers in the future.

**Table 2:** Summary of structural optimization of interlayers in LSBs.

Interlayer material	Sulfur content [wt%]	Sulfur loading [mg cm^−2^]	Rate [C]	Cycle number	First discharge capacity [mAh g^−1^]	Discharge capacity after cycling [mAh g^−1^]	Refs.
SWCNTAs	N/A	10.35	0.1	60	N/A	559	[55]
HC‐In	7057	2.2	0.5	100	935	604	[56]
PPC film	75	3.5	0.5	300	849.9	458.1	[57]
Cu_2_(CuTCPP)	64	2.0	1	900	850	604	[58]
CON‐TFSI	N/A	0.6	0.2	500	981.9	481.4	[59]
CNT	70	N/A	0.1	100	1660.4	958.3	[60]
NF	N/A	3.1	0.2	220	918	685	[61]
3DHCI‐19	95	3.3	0.2	200	692	663	[62]
HCF‐800	N/A	2.23	0.1	100	612	502	[63]
MOF‐808/CNT	N/A	2.0	0.2	100	1272	1134.6	[64]
DMTA‐COF	80	2.0	1	500	1068	621	[65]

## 4 Lithiophilicity Microenvironment Engineering

Lithiophilicity microenvironmental engineering is a strategy to optimize the overall performance of LSBs through chemical regulation. In contrast to the physical design of structural engineering, it focuses on enhancing the chemical affinity of material surfaces toward lithium species, creating a lithophilic environment that promotes the intercalation‐deintercalation of Li‐ion and achieves the chemical immobilization of LiPSs. Therefore, many studies have been devoted to constructing highly active lithophilic sites, and have made much progress in this field.

### 4.1 Pristine Metal‐Free Polar Materials

**Figure 5:**
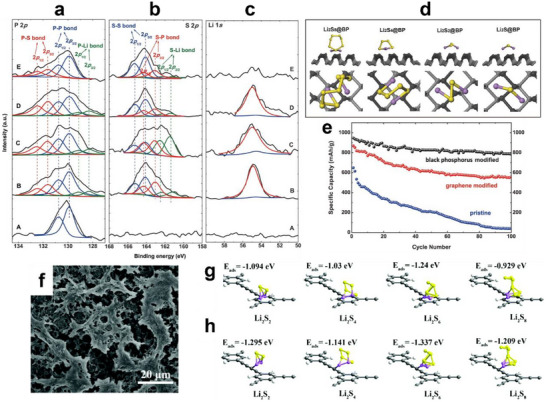
a) P 2p, b) S 2p, and c) Li 1s XPS spectra of the BP‐modified separators. d) The molecular models of the interaction between BP and Li_2_S_8_, Li_2_S_4_, Li_2_S_2_, and Li_2_S calculated via first‐principles. e) Cycling performance of the three LSBs after 100 cycles at 0.4 A g^−1^. (a–e) Reproduced with permission.^[^ ^66^ ^]^ Copyright 2016, Wiley‐VCH. f) SEM image of HsGDY. Theoretical simulations the Li_2_S_n_ (n = 2, 4, 6, 8) molecule adsorbed on HsGDY flakes. g) The Li_2_S_n_ molecule adsorbed on the sp^2^ hybridized carbon of HsGDY. h) The Li_2_S_n_ molecule adsorbed on the sp hybridized carbon of HsGDY. (f–h) Reproduced with permission.^[^ ^69^ ^]^ Copyright 2021, Royal Society of Chemistry.

Pristine metal‐free polar materials possess great potential in enhancing the lithiophilicity of LSBs because they can interact with lithium species through intrinsic polar chemical bonds or electron‐rich structures without additional modifications. Therefore, many inorganic polar materials have been introduced into LSBs to adsorb LiPSs and inhibit the shuttle effect. Sun et al. reported a functional separator coated with black‐phosphorus (BP) nanoflakes.^[^ ^66^ ^]^ The high electrical conductivity (≈300 S m^−1^)^[^ ^67^ ^]^ and ultra‐fast Li‐ion diffusion properties (10^4^ times faster than graphene)^[^ ^68^ ^]^ of BP could promote Li‐ion transport and enhance redox kinetics, thereby minimizing capacity loss. Furthermore, the BP coating layer not only physically adsorbed LiPSs but also chemically bonded with them through P‐S and P‐Li interactions (**Figure** **5**a–c). First‐principles calculation (Figure 5d) revealed that the bonding energies between BP and LiPSs are much stronger than on graphene and polar polymers. This indicated that BP generated robust chemisorption, thus effectively inhibiting LiPSs diffusion and shuttling. Benefiting from these attributes, the LSBs with the BP separator exhibited a high initial discharge capacity of 930 at 0.4 A g^−1^ and retained the capacity retention of 86% after 100 cycles (Figure 5e).

In addition to the above‐mentioned inorganic materials, polar organic polymers with electron‐rich structures have been employed to construct lithophilic environments. Kong et al. designed a hydrogen‐substituted graphdiyne (HsGDY)/graphene (Gra) composite interlayer for LSBs.^[^ ^69^ ^]^ The HsGDY/Gra interlayer, with an ultrahigh specific surface area (2184 m^2^ g^−1^) and hierarchical pores, physically trapped LiPSs while allowing rapid Li‐ion migration (Figure 5f). More importantly, the acetylenic bonds in HsGDY chemically anchor the lithium species of the LiPSs via strong Li‐C interactions (Figure 5g,h). This dual adsorption mechanism effectively restrained the shuttle effect and promoted LiPSs conversion kinetics.

### 4.2 Non‐Metal Heteroatom Doping Modifications

Heteroatom doping modification is an effective strategy for constructing lithiophilic microenvironments. According to the Lewis acid‐base theory, heteroatoms with lone electron pairs (such as N, S, P) can be regarded as Lewis bases,^[^ ^70^ ^]^ coordinating with the positively charged lithium ions in LiPSs through dipole‐dipole interactions, thereby chemically anchoring LiPSs and mitigating the inevitable shuttle effect in LSBs.^[^ ^71^, ^72^ ^]^ Currently, non‐metal heteroatoms are mainly doped into carbon‐based materials to optimize sulfur hosts and interlayers in LSBs.

#### 4.2.1 Single‐Atom Doping Modifications

N‐doping is one of the most widely used modification methods nowadays since the abundant N sites not only improve the conductivity to accelerate Li‐ion transfer but also alter the electronic structure of carbon materials to enhance the chemical adsorption of LiPSs. Huang's group developed N‐doped hollow porous carbon spheres (NHPCS) as a sulfur host for LSBs through a simple template strategy.^[^ ^73^ ^]^ The NHPCS, with a high specific surface area (1526 m^2^ g^−1^) and hierarchical porous structure (mesopore sizes of 2.5/15 nm), increased the sulfur loading accommodated the volume expansion of sulfur during discharge, and physically confined LiPSs diffusion. Additionally, the doped N atoms provided NHPCS with abundant active sites, which could effectively anchor dissolved LiPSs through strong chemical interactions, thus inhibiting the shuttle effect.

S‐doping has also been proven to be effective in enhancing the polarity of carbon matrices and promoting the immobilization of LiPSs. Recently, Ma et al. constructed S‐doped mesoporous graphene (SMG) using MgSO_4_ as sulfur source and MgO as a template by a fluidized‐bed chemical vapor deposition (CVD) method and employed it as a separator modifier for LSBs.^[^ ^74^ ^]^ The SMG featuring a large specific surface area (1705 m^2^ g^−1^) and abundant pore structures (pore sizes less than 10 nm) could provide sufficient physical confinement for LiPSs and hinder their migration to the anode. Moreover, the doped sulfur existing in the form of C─S─C and C═S bonds, enhanced the polarity of carbon materials and formed stronger interactions with LiPSs. Density functional theory (DFT) calculations and UV–vis adsorption experiments demonstrate that SMG had stronger adsorption capacity toward LiPSs than undoped mesoporous graphene (MG), which effectively captured dissolved polysulfides and improved the utilization of active materials. Therefore, the LSBs with SMG/PP separator yielded an initial discharge capacity of 894.21 mAh g^−1^ at 1 C and delivered 601.29 mAh g^−1^ after 300 cycles.

**Figure 6:**
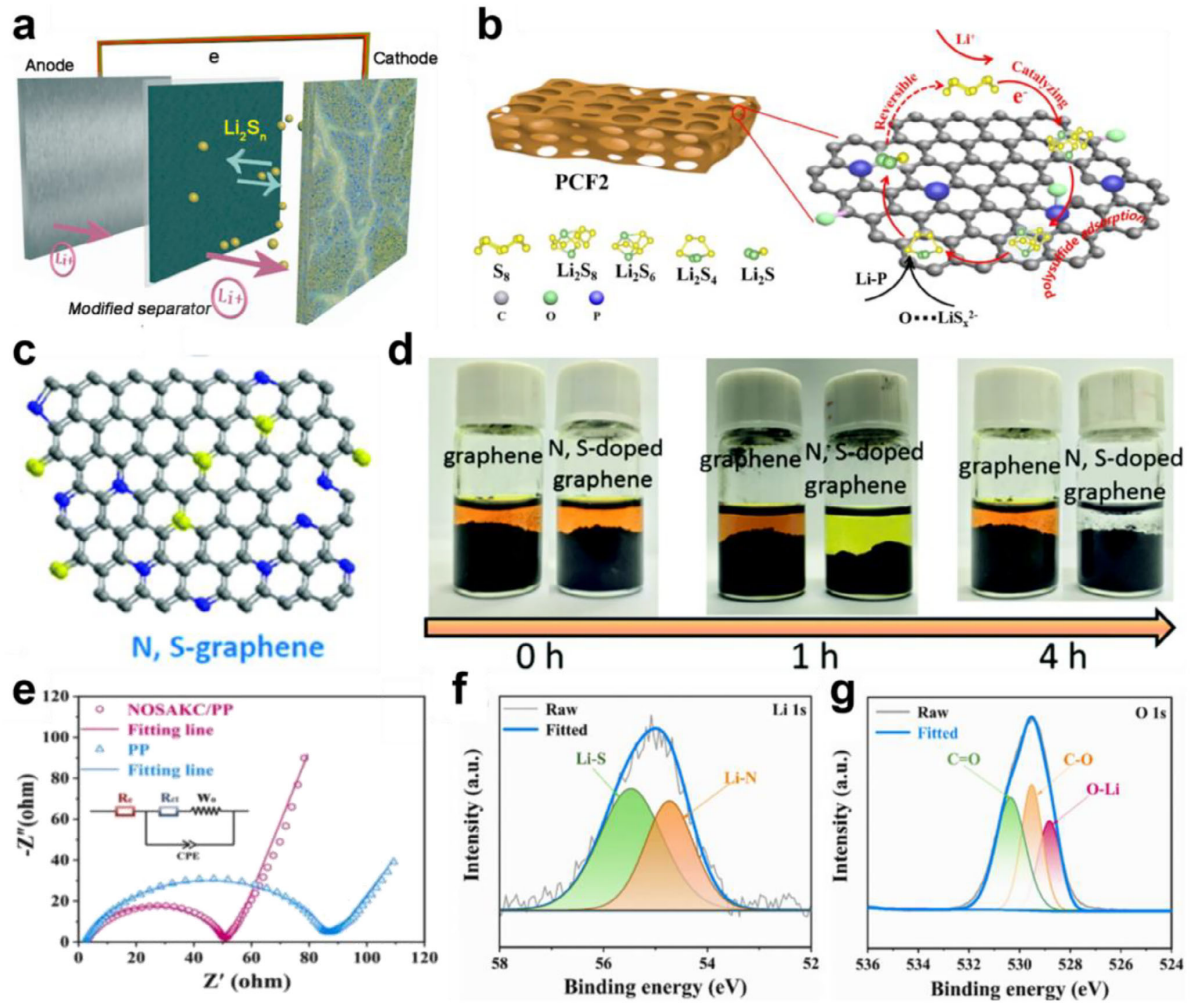
a) Schematic illustration of LSB decorated with modified separator. b) Mechanism diagram of the reaction between sulfur species. (a,b) Reproduced with permission.^[^ ^75^ ^]^ Copyright 2024, Elsevier. c) The schematic illustration of the synthesis of N, S‐doped graphene. d) Photograph of the polysulfide solution after exposure to different adsorbers: graphene and N, S‐doped graphene. (c,d) Reproduced with permission.^[^ ^76^ ^]^ Copyright 2022, Royal Society of Chemistry. e) Electrochemical Impendance spectroscopy (EIS) plots of different separators before cycling. High‐resolution XPS spectra of NOSAKC/PP after cycling: f) Li 1 s, g) O 1 s. (e–g) Reproduced with permission.^[^ ^77^ ^]^ Copyright 2025, Elsevier.

Besides the N atom and S atom, P can be introduced into carbon materials. Notably, P‐doping can provide a dual mechanism of chemical adsorption and catalytic conversion for LiPSs. For example, Zou et al. prepared P‐doped carbon foam (PCF) via a facile template‐free method as sulfur hosts (**Figure** **6**a).^[^ ^75^ ^]^ The PCF possessed a hierarchical porous structure, which encapsulated more sulfur and improved Li‐ion diffusion behaviors. In addition, doped phosphorus atoms endowed PCF with lithophilic properties, promoting the bonding between Li species and polar atoms at the interface, thereby enhancing the adsorption and limiting LiPSs migration (Figure 6b). More importantly, compared to N‐ and S‐doping, the P‐doping exhibited a positive catalytic conversion effect on LiPSs, which effectively reduced the nucleation energy barrier and accelerated sulfur reaction kinetics.

#### 4.2.2 Multi‐Atom Doping Modifications

Multi‐atom doping modifications play a significant role in realizing lithiophilicity for LSBs. The synergistic effect of multi‐atom doping generates more active sites and stronger chemisorption capabilities, which further inhibits LiPSs shuttling and maximizes the electrochemical performance of LSBs. Among them, dual‐atom doping has been extensively investigated to deal with the shuttle effect. Li et al. synthesized N, S‐doped graphene with high nitrogen and sulfur contents by the self‐assembly strategy and subsequent calcination processes and applied in the LSBs electrode (Figure 6c).^[^ ^76^ ^]^ N, S‐doped graphene with randomly oriented sheet‐like structures and high electrical conductivity provided fast pathways for Li‐ion transport, effectively reduced interfacial resistance, and promoted the electrochemical reaction kinetics. In addition, the high doping of N and S formed abundant active sites such as pyridinic N (398.4 eV), graphitic N (401.2 eV), C─S─C bonds (163.9 eV) as well as S‐O bonds (165.2 eV). Thus, N, S‐doped graphene achieved strong chemisorption of LiPSs through the interaction of Li‐N and S‐S bonds, suppressing the shuttle effect (Figure 6d). As a result, the cathode with the N, S‐doped graphene exhibited excellent rate performance and outstanding cycling stability.

Recently, researchers have also proposed the incorporation of three distinct atoms into carbon‐based materials. For example, Chen et al. prepared N, O, S tri‐doped carbon (NOSAKC) through pyrolysis of C_4_H_4_KNO_4_S and applied it to modify the commercial separator for LSBs.^[^ ^77^ ^]^ The NOASKC, with a 3D porous honeycomb‐like structure, hierarchical pores, and a large specific surface area (336.7 m^2^ g^−1^), could physically confine LiPSs to the cathode side while facilitating electrolyte penetration and Li‐ion migration (Figure 6e). Furthermore, high conductivity and good graphitization of NOSAKC reduced the interfacial resistance (52.6 Ω), significantly accelerating the charge transfer (Figure 6f). Most importantly, the uniform doping of N, O, and S atoms provided rich adsorption/conversion sites for NOSAKC, enabling the chemical anchoring of LiPSs through Li─N bonds (54.9 eV), Li─S bonds (55.6 eV) and O─Li bonds (528.6 eV), thereby suppressing the shuttle effect (Figure 6g).

### 4.3 Polar Functional Groups Modifications

The incorporation of polar functional groups on the surface or in the structure of materials is considered a promising microenvironmental engineering for lithiophilicity. These characteristic groups, including amino (‐NH_2_), hydroxyl (‐OH), carboxyl (‐COOH), and so on, typically possess lone electron pairs or strong electronegativity, enabling them to engage in robust chemical interactions with lithium species, thus effectively anchoring LiPSs and alleviating the severe shuttle effect. Recently, modifications with polar functional groups have been widely explored in LSBs. For example, Liang et al. designed a sulfur host material (HOCNTs) by introducing oxygen‐containing functional groups into thin‐wall porous amorphous carbon nanotubes (HCNTs) using H_2_O_2_ treatment.^[^ ^78^ ^]^ HOCNTs exhibited a large specific surface area (465 m^2^ g^−1^), hierarchical porosity, and a unique thin‐walled structure, which facilitated the penetration and dispersion of sulfur, avoiding direct contact between active materials and the electrolyte and thus physically alleviating the dissolution and diffusion of LiPSs. Furthermore, the abundant O‐H/C‐O groups enhanced the chemical adsorption capability of HOCNTs toward LiPSs, effectively inhibiting the shuttle effect.

**Figure 7:**
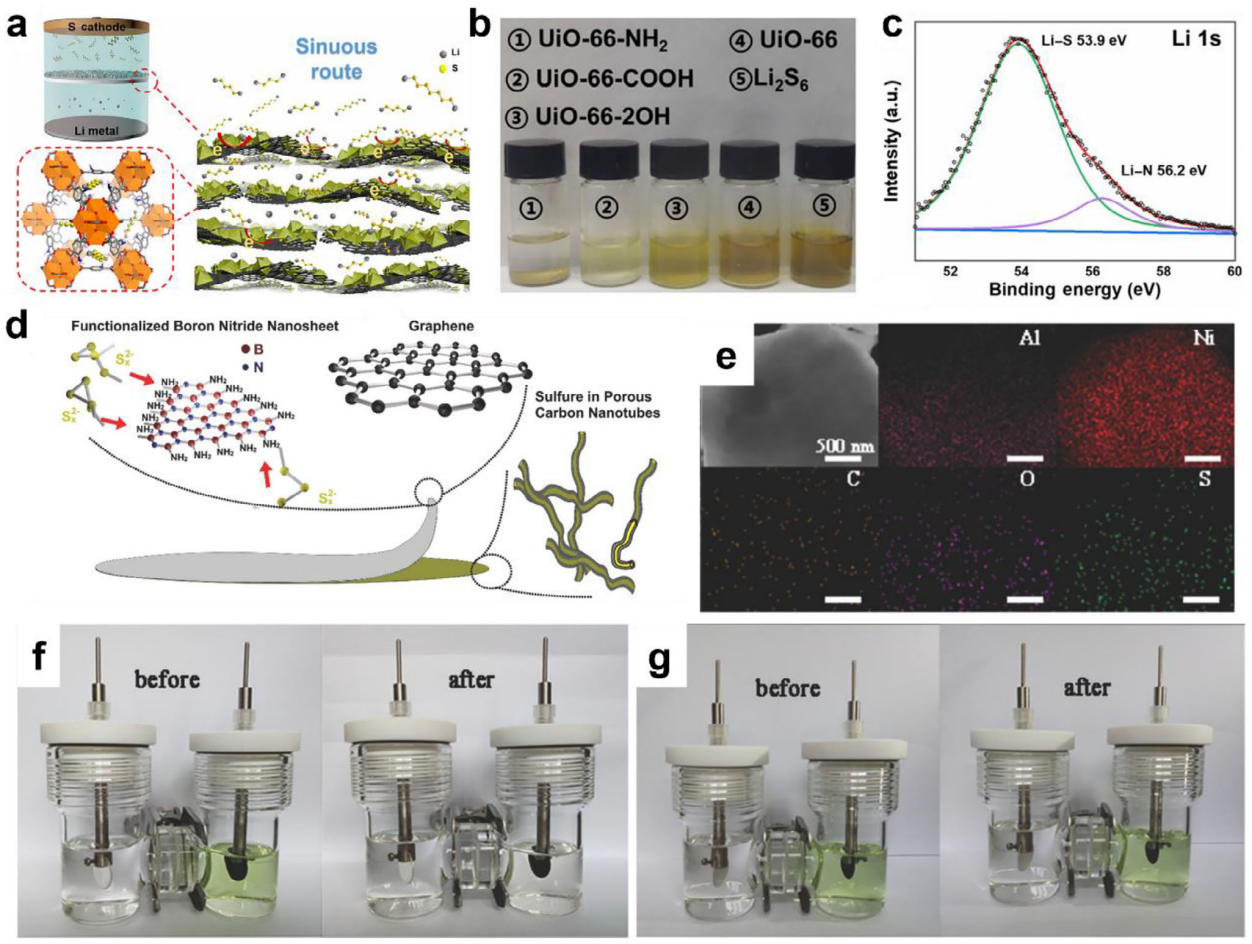
a) Schematic illustration for the design of both chemical interaction and microstructure of interlayer constructed with MOF and graphene. b) The digital photos of Li_2_S_6_/DME/DOL before and after adsorption with different functionalized MOFs. c) Li 1s XPS spectrum of Li_2_S_6_@UiO66‐NH_2_. (a–c) Reproduced with permission.^[^ ^79^ ^]^ Copyright 2021, Springer Nature. d) Schematic configuration of LSBs with an FBN/G interlayer.^[^ ^81^ ^]^ Copyright 2017, Wiley‐VCH. e) STEM and corresponding elemental mapping of CNTs/Gra/Al_3_Ni_2_ electrode after ten cycles of cyclic voltammetry (CV) test. Digital photograph of the H‐type simulation electrolytic cell CV tests. f) CNTs/Gra/Al_3_Ni_2_ cathode before and after 150 CV cycles. g) CNTs/Gra cathode before and after 150 CV cycles. (e–g) Reproduced with permission.^[^ ^82^ ^]^ Copyright 2018, Wiley‐VCH.

Beside the aforementioned materials, framework materials (such as MOFs and COFs) have attracted considerable attention as matrices for functional group modifications, which is mainly attributed to their intrinsic merits, including exceptional specific surface area and precisely tunable porous structures. Upon functionalization, these frameworks can provide additional active sites for the adsorption of LiPSs. Recently, Guo et al. established an ordered (UiO‐66‐NH_2_@graphene) interlayer through the assembly of amino‐functionalized MOFs on graphene (**Figure** **7**a).^[^ ^79^ ^]^ UiO‐66‐NH_2_@graphene possessed an ordered layered structure with MOF particles uniformly dispersed on graphite sheets, which could form tortuous channels to physically mitigate LiPSs migration without impairing Li‐ion transport. More importantly, the abundant amino functional groups (‐NH_2_) on the interlayer strongly adsorb lithium species in LiPSs through polar interactions (Figure 7b,c), thereby effectively suppressing the shuttle effect. Therefore, the LSBs with the UiO‐66‐NH_2_@graphene interlayer attained a high discharge capacity of 890 mAh g^−1^ at 1 C. To further improve the electrochemical performance of LSBs, multiple functional groups can be introduced into framework materials to achieve synergistic effects on the adsorption and catalytic conversion of LiPSs. Sun et al. prepared a silanol‐branched covalent organic framework (TPAS‐TPB‐COF) via a solvothermal method.^[^ ^80^ ^]^ TPAS‐TPB‐COF with a large specific surface area (417 m^2^ g^−1^) and microporous channels (1.35 nm) offered enough space to load sulfur (71 wt.%) and restrain LiPSs diffusion. TPAS with silanol groups (Si‐OH) endowed the COF with strong polarity (ζ‐potential: −43.8 mV), enabling the anchoring of LiPSs through hydrogen bonding and chemical adsorption. Meanwhile, TPB provided abundant redox active sites, such as hydroxyl (‐OH) and carbonyl (C═O) groups, which catalyzed the rapid conversion of LiPSs, significantly enhancing the reaction kinetics of LSBs. Huang's group fabricated an interlayer for LSBs by applying a thin coating of graphene and ─NH_2_ functionalized boron nitride nanosheets (FBN) to the cathode (Figure 7d).^[^ ^81^ ^]^ The interlayer not only reduces charge transfer resistance but also alleviates the shuttling issue. The ─NH_2_ group on FBN serve as sites that attract LiPSs through electrostatic interactions. As a result, LSBs with FBN/graphene interlayer demonstrate an enhanced capacity of 560 mAh g^−1^ at 3 C, and the rate of cyclic capacity decay is lowered to 0.0037% per cycle.

## 5 Sulfiphilicity Microenvironment Engineering

Unlike lithiophilicity optimization anchoring lithium species, sulfiphilicity microenvironment engineering is dedicated to boosting the chemical affinity between host materials or interlayers and sulfur species, constructing active interfaces with highly efficient adsorption and catalytic functions, thus realizing precise anchoring and rapid conversion of LiPSs. Therefore, it plays a critical role in suppressing the shuttle effect of LiPSs, improving sluggish redox kinetics, and enhancing the energy density of LSBs. Recently, research on this engineering mainly focuses on metal center regulation and organosulfur copolymerization regulation, which will be introduced in the following sections.

### 5.1 Metal Centers Regulation

Metal centers regulation is a widely recognized strategy for constructing sulfiphilicity microenvironments. The introduction of metal centers with sulfur affinity can achieve the chemical adsorption of LiPSs, effectively alleviating the shuttle effect. More importantly, some metal centers possess the dual functions of chemical adsorption and catalytic conversion, which restricts the diffusion of LiPSs while accelerating the reaction kinetics of sulfur. Consequently, metal center regulation has been extensively studied and applied in LSBs. According to the form of metal centers, research can be mainly divided into the following three aspects: 1) metal centers in metals or metal‐alloys; 2) metal centers in transition metal compounds (TMCs); and 3) metal centers in MOFs.

#### 5.1.1 Metal Centers in Metals or Metal‐Alloys

Metals have been widely applied in LSBs to suppress the shuttle effect of LiPSs due to their inherent electronic conductivity and catalytic activity. Recently, Huang et al. designed a nickel nanoparticle‐decorated and nitrogen‐doped carbonized bacterial cellulose (Ni‐NCBC) interlayer by initial coating and subsequent carbonization process.^[^ ^82^ ^]^ The Ni‐NCBC interlayer with highly interconnected conductive networks exhibited a macropores structure (100 nm), which facilitated Li‐ion transport and physically confined LiPSs diffusion. Additionally, through DFT calculations confirmed, N‐doping and Ni nanoparticles endowed Ni‐NCBC with strong chemical adsorption of LiPSs, effectively suppressing the shuttle effect. More importantly, the embedded Ni nanoparticles reduced the energy barrier of LiPSs conversion reactions by electrocatalysis, thereby significantly improving the redox kinetics.

Notably, metals are usually susceptible to corrosion by polysulfides or electrolytes due to their poor structural stability, which leads to a significant decrease in conductivity and adsorption properties. Therefore, metal alloys with good mechanical stability have been introduced into LSBs for rationally constructing sulfiphilicity microenvironments. Huang's group developed a 3D CNTs/Gra‐S‐Al_3_Ni_2_ cathode by integrating carbon nanotubes (CNTs), graphene (Gra), sulfur (S), and Al_3_Ni_2_ alloy for LSBs.^[^ ^83^ ^]^ The 3D CNT/Gra network improved electrical conductivity and reduced ion diffusion distances. Furthermore, the Al_3_Ni_2_ alloy played a dual role in LSBs. Specifically, Al facilitated Li‐ion transmission and provided structural stability to the cathode, while Ni effectively inhibited the shuttle effect by catalyzing the rapid conversion and strong chemical adsorption of LiPSs (Figure 7e–g). Benefiting from these advantages, the CNTs/Gra‐S‐Al_3_Ni_2_ cathode displayed excellent rate performance and outstanding cycling stability.

#### 5.1.2 Metal Centers in Transition Metal Compounds

Transition metal compounds (TMCs) have demonstrated great potential in sulfiphilicity microenvironment engineering due to their tunable electronic structures and abundant active sites. Specifically, transition metal centers with partially filled d‐orbitals can effectively anchor LiPSs while accelerating redox kinetics. Meanwhile, non‐metallic ligands can modulate the electron distribution of transition metals and enhance the overall structural stability. As a result, TMCs, particularly transition metal oxides (TMOs), sulfides (TMSs), selenides (TMSes), nitrides (TMNs), and phosphides (TMPs), have been devoted to constructing sulfiphilic microenvironments in LSBs.

**Figure 8:**
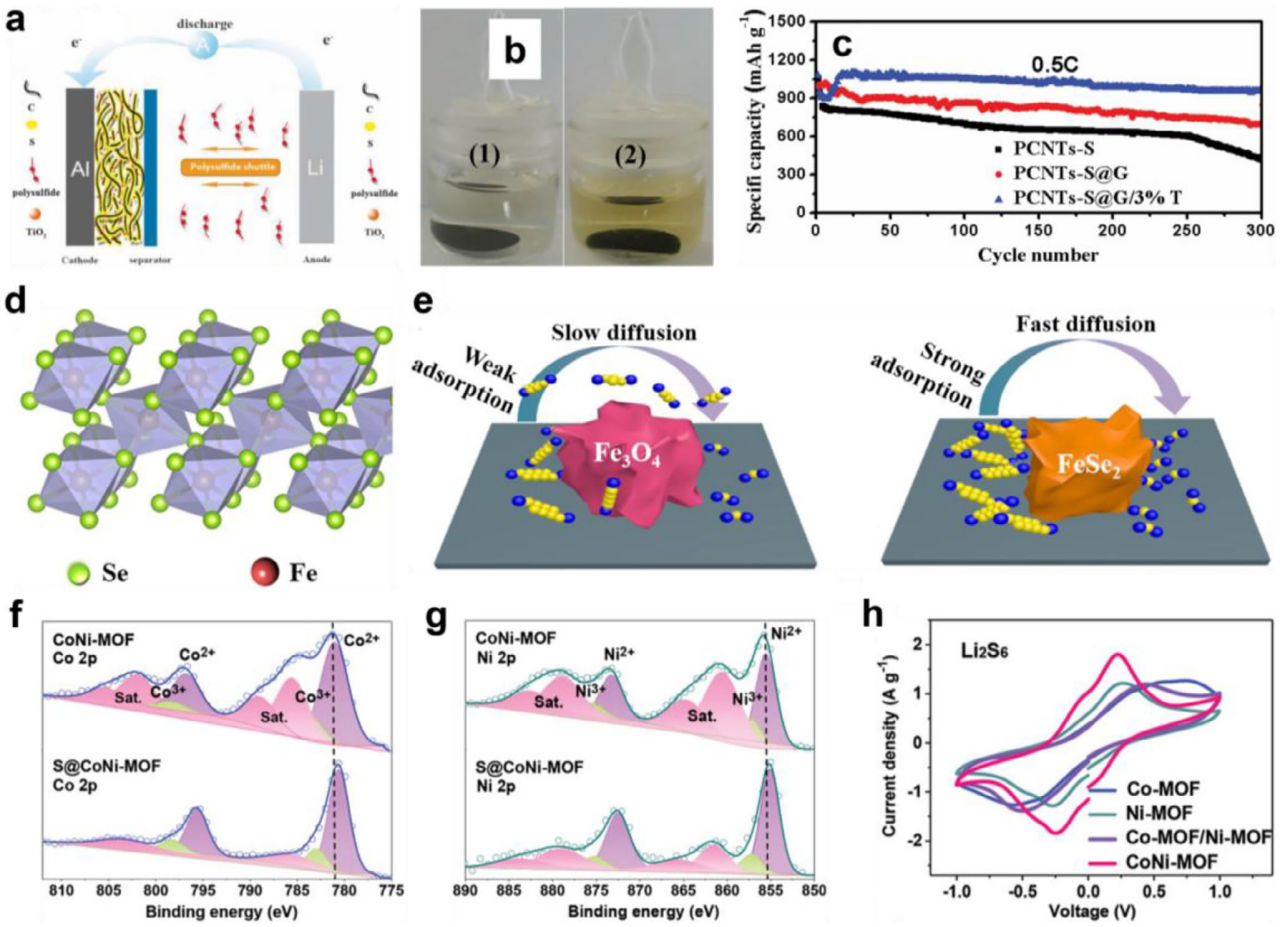
a) Schematic of electrode configuration for Li‐S battery with a graphene/TiO_2_ coating film. b) Typical colors of electrolyte for the PCNTs‐S@G/3%T (1) and PCNTs‐S (2) cathodes after 300 cycles in sealed vials. c) cycling stability of PCNTs‐S, PCNTs‐S@G, PCNTs‐S@G/3%T cathodes at 0.5 C, the current density of 0.86 mA cm^−2^. (a–c) Reproduced with permission.^[^ ^84^ ^]^ Copyright 2015, Wiley‐VCH. d) Crystal structure of orthorhombic FeSe_2_. e) The comparison diagram of LiPSs adsorption and diffusion on Fe_3_O_4_@C and FeSe_2_@C matrix. (d,e) Reproduced with permission.^[^ ^86^ ^]^ Copyright 2021, Elsevier. High‐resolution XPS spectra of f) Co 2p and g) Ni 2p of CoNi‐MOF and S@CoNi‐MOF composites. h) CV curves of Li_2_S_6_ symmetrical cells with Co‐MOF, Ni‐MOF, CoNi‐MOF, and Co‐MOF/Ni‐MOF loaded CP. (g–i) Reproduced with permission.^[^ ^90^ ^]^ Copyright 2021, Wiley‐VCH.

TMOs have attracted much attention since they can effectively adsorb LiPSs through Lewis acid‐base interactions. Huang's group designed a lightweight TiO_2_/graphene interlayer (7.8 wt.% of the cathode) via a facile coating method for LSBs (**Figure** **8**a).^[^ ^84^ ^]^ The interconnected graphene enhanced electron/ion transport and provided a physical barrier for confining LiPSs. Meanwhile, the TiO_2_ with sulfiphilic properties chemically anchored dissolved LiPSs through strong S‐Ti‐O electrostatic interactions and Ti‐S bonding, thus effectively inhibiting the shuttle effect and promoting the redox reaction (Figure 8b). As a result, the LSBs with the TiO_2_/graphene interlayer exhibited a high initial discharge capacity of 1050 mAh g^−1^ at 0.5 C and yielded 1040 mAh g^−1^ capacity after 300 cycles (Figure 8c), which is much higher than C‐S cathodes without TiO_2_.

TMSs and TMSes are widely explored in the design of sulfiphilic microenvironments for LSBs due to their excellent catalytic activity. He et al. prepared 1T‐rich MoS_2_@PC composites by anchoring 1T‐rich MoS_2_ on a conductive porous carbon via a two‐step hydrothermal‐carbonization method.^[^ ^85^ ^]^ The conductive carbon skeleton uniformly dispersed 1T‐phase MoS_2_ nanosheets (≈100 nm) while facilitating rapid electron/ion transport. Moreover, 1T‐rich MoS_2_ nanosheets endowed the matrix surface with good electrical conductivity and abundant sulfiphilic sites, which not only enhanced the adsorption capacity of LiPSs but also accelerated the liquid‐liquid and liquid‐solid transformations of LiPSs. Such a dual property could effectively inhibit the severe shuttling effect and improve the sluggish redox kinetics. Furthermore, Sun et al. constructed FeSe_2_ nanoparticles encapsulated in carbon nanoboxes (FeSe_2_@C) through the selenide reaction of yolk‐shelled Fe_3_O_4_@C (Figure 8d).^[^ ^86^ ^]^ Both DFT calculations and experimental results showed that FeSe_2_@C exhibits stronger chemical interactions with LiPSs, along with enhanced conductivity and catalytic activity compared to Fe_3_O_4_@C. These properties mitigated the shuttle effect and promoted redox reactions in LSBs (Figure 8e). The LSBs with S/FeSe_2_@C cathodes demonstrated excellent cycling stability, showing an ultra‐low capacity decay of 0.04% per cycle after 700 cycles at 1 C.

TMNs and TMPs have been found to effectively suppress LiPSs shuttling because of their good thermal stability and adsorption‐catalytic synergistic effects. Peng et al. synthesized TiN nanoflakes with exposed (001) facets by using Ti_3_C_2_ MXene as the precursor.^[^ ^87^ ^]^ These nanoflakes acted as bidirectional electrocatalysts for both reduction and oxidation reactions in LSBs. The (001) facet‐dominated TiN nanoflakes demonstrated strong adsorption of soluble LiPSs, which is crucial for improving the performance of LSBs. This adsorption promoted the conversion of soluble LiPSs to Li_2_S_2_/Li_2_S during discharge and reduced the delithiation barrier of Li_2_S during charging. As a result, TiN‐based sulfur hosts in LSBs exhibited remarkable electrochemical properties. Moreover, Sun et al. prepared a multifunctional sulfur host of yolk‐shelled Fe_2_N@C nanoboxes (Fe_2_N@C NBs) through a process combining etching and nitridation.^[^ ^88^ ^]^ The structure featured a conductive carbon shell that facilitated fast electron and ion transport, along with a polar Fe_2_N core that enhanced chemisorption and catalysis of LiPSs. This design improved sulfur retention, mitigated the shuttling effect, and accommodated volume expansion during cycling. The Fe_2_N@C NBs host offered high sulfur content, excellent capacity, rate capability, and long‐term stability.

Zhang et al. constructed a 3D porous FeP/rGO (FPG) microspheres via spray‐drying and phosphorization treatments and applied them as both the cathode and interlayer for LSBs.^[^ ^89^ ^]^ The 3D porous FeP/rGO with numerous large voids (250 to 350 nm) and conductive network allowed for high sulfur loading and fast electron transport. FeP nanoparticles effectively anchored sulfur species through the robust interactions of Li‐P and S‐Fe bonds, realizing the strong chemisorption of LiPSs (adsorption energy: −4.21 to −1.975 eV). In addition, FeP promoted the transformation of LiPSs through electrochemical catalysis, further suppressing the shuttle effect.

#### 5.1.3 Metal Centers in MOFs

MOFs have been widely used to construct sulfiphilic microenvironments due to their ordered porous structures, open polar sites, and excellent metal catalytic centers. Meng et al. synthesized ultrathin 2D bimetal CoNi‐MOF nanosheets via a sonochemical method and applied them with CNTs as a functional interlayer for LSBs.^[^ ^90^ ^]^ The CoNi‐MOF nanosheets possessed a large specific surface area (49.66 m^2^ g^−1^) and micro‐mesoporous structure (pore size: 8.4 nm), which provided abundant exposed active sites and physically confined LiPSs diffusion. More importantly, the Ni and Co metal sites in 2D MOFs had strong chemical interactions with sulfur species, which formed Ni‐S bonds (Figure 8g) and Co‐S bonds (Figure 8g) and enhanced adsorption capacity for LiPSs. Meanwhile, the bimetallic synergistic effect optimized the coordination environment of the active sites, improving catalytic performance and thus accelerating the liquid‐liquid and liquid‐solid conversion kinetics of LiPSs (Figure 8h).

### 5.2 Organosulfur Copolymerization Regulation

**Figure 9:**
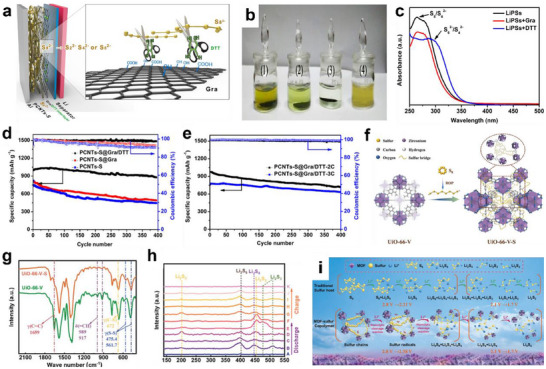
a) Schematic of electrode configuration for Li‐S battery with Gra/DTT interlayer. b) Visualized color change after PCNTs‐S, PCNTs‐S@Gra, PCNTs‐S@Gra/DTT cathodes immersing in (1), (2), (3) solution for 6 h, respectively, (4) as reference. c) UV‐vis absorption spectra of the solution after soaking fresh PCNTs‐S, PCNTs‐S@Gra, and PCNTs‐S@Gra/DTT cathodes. d) Cycling stability of PCNTs‐S@Gra/DTT, PCNTs‐S@Gra, and PCNTs‐S cathodes at 1 C. e) Cycling performance of PCNTs‐S@Gra/DTT cathode at 2 and 3 C. (a‐e) Reproduced with permission.^[^ ^91^ ^]^ Copyright 2017, American Chemical Society. f) The synthesis route of UiO‐66‐V‐S. g) FT‐IR spectra of UiO‐66‐V and UiO‐66‐V‐S. h) In situ Raman spectra of UiO‐66‐V‐S cathode. i) Proposed transformation processes of sulfur species in polymerized UiO‐66‐V‐S cathode. (f–i) Reproduced with permission.^[^ ^92^ ^]^ Copyright 2022, Wiley‐VCH.

Organosulfur copolymerization regulation can construct an ideal sulfiphilic microenvironment by introducing organic materials with sulfur‐affinity groups (‐SH, ─C═C, ─C≡N) into LSBs, achieving covalent or dynamic non‐covalent immobilization of LiPSs. Organic small molecules are regarded as promising materials in organosulfur copolymerization regulation because they can achieve precise anchoring and efficient conversion of LiPSs through sulfiphilic functional groups. Notably, many organic small molecules suffer from poor conductivity, so they need to be combined with conductive matrices for application in LSBs. Huang's group designed a lightweight graphene/dithiothreitol (Gra/DTT) interlayer and subsequently coated it on a porous carbon nanotubes/S cathode (PCNTs‐S) for LSBs (**Figure** **9**a).^[^ ^91^ ^]^ DTT, a sulfiphilic organic reagent, cleaved the ─S‐S‐ bonds in LiPSs through biochemical reactions under mild conditions, converting them into short‐chain sulfides such as Li_2_S_2_ and Li_2_S (Figure 9b,c). Such a targeted polysulfide‐scission effect could minimize the accumulation of dissolved LiPSs in the electrolyte, effectively suppressing the shuttle effect. As a result, the PCNTs‐S@Gra/DTT cathode delivered high initial discharge capacities of 997, 975, and 762 mAh g^−1^ at 1 C, 2 C, and 3 C, respectively (Figure 9d,e).

Using organic framework materials containing sulfiphilic groups is also a significant strategy for organosulfur copolymerization regulation in LSBs. Recently, Huang's group prepared a MOF‐sulfur copolymer (CNT@UiO‐66‐V‐S) via copolymerizing sulfur chains with vinyl‐functionalized MOFs through the inverse vulcanization and applied it as the cathode in LSBs (Figure 9f).^[^ ^92^ ^]^ Sulfur was uniformly distributed within the MOF pores in a polymeric state, which mitigated the volume expansion of crystalline sulfur and ensured the structural stability of the cathode. Crucially, the vinyl groups in the MOF formed C‐S bonds with the polymeric sulfur (Figure 9g), providing active sites for the targeted anchoring of polysulfides, effectively limiting their dissolution and diffusion. Furthermore, the polymeric sulfur chains shorten the reaction pathway through a radical reaction mechanism (Figure 9h,i), accelerating the redox kinetics of the sulfur cathode. Therefore, the CNT@UiO‐66‐V‐S cathode exhibited excellent rate performance and cycling stability, achieving a high capacity of 609 mAh g^−1^ even after 1000 cycles at 1 C.

## 6 Lithiophilicity‐Sulfiphilicity Microenvironment Engineering

Lithiophilicity‐sulfiphilicity microenvironment engineering is an innovative strategy to optimize the overall performance of LSBs by simultaneously regulating the adsorption, diffusion, and reaction behaviors of both lithium and sulfur species. Different from a single microenvironment engineering, it integrates dual‐functional active sites with lithiophilicity and sulfiphilicity, which not only promotes the transport of Li‐ion but also achieves the chemical adsorption and catalytic conversion of LiPSs. Therefore, constructing a lithiophilic‐sulfiphilic multifunctional microenvironment is crucial in the design of high‐performance LSBs. Currently, many studies in this field can be summarized and explored in the following two aspects: 1) Traditional catalysts structure regulation; 2) MOFs catalysts structure regulation.

### 6.1 Traditional Catalysts Structure Regulation

Traditional catalysts structure regulation is a crucial strategy for creating lithiophilic‐sulfiphilic microenvironments, as it can alter the physical and chemical properties of materials by introducing or modulating functional sites, thereby optimizing the overall performance of LSBs. Recently, research on traditional catalysts structure regulation has mainly focused on these five aspects: 1) heterostructure construction; 2) multicomponent incorporation; 3) vacancy; 4) doping; 5) coordination environmental of metal single atom catalysts (SACs), which will be described in the following sections.

#### 6.1.1 Heterostructure Construction

Heterostructure construction is considered an effective strategy for realizing a synergistic lithiophilic‐sulfiphilic microenvironment in LSBs. Specifically, heterostructure integrates two or more components into lithiophilic/sulfiphilic dual‐active domains through interfacial coupling effects, thereby enhancing the chemical adsorption of LiPSs and modulating the transport behavior of Li‐ion. Such a design not only suppresses the shuttle effect of LiPSs but also guides the uniform deposition of Li‐ion, which significantly improves the cycling stability and redox kinetics of LSBs.

**Figure 10:**
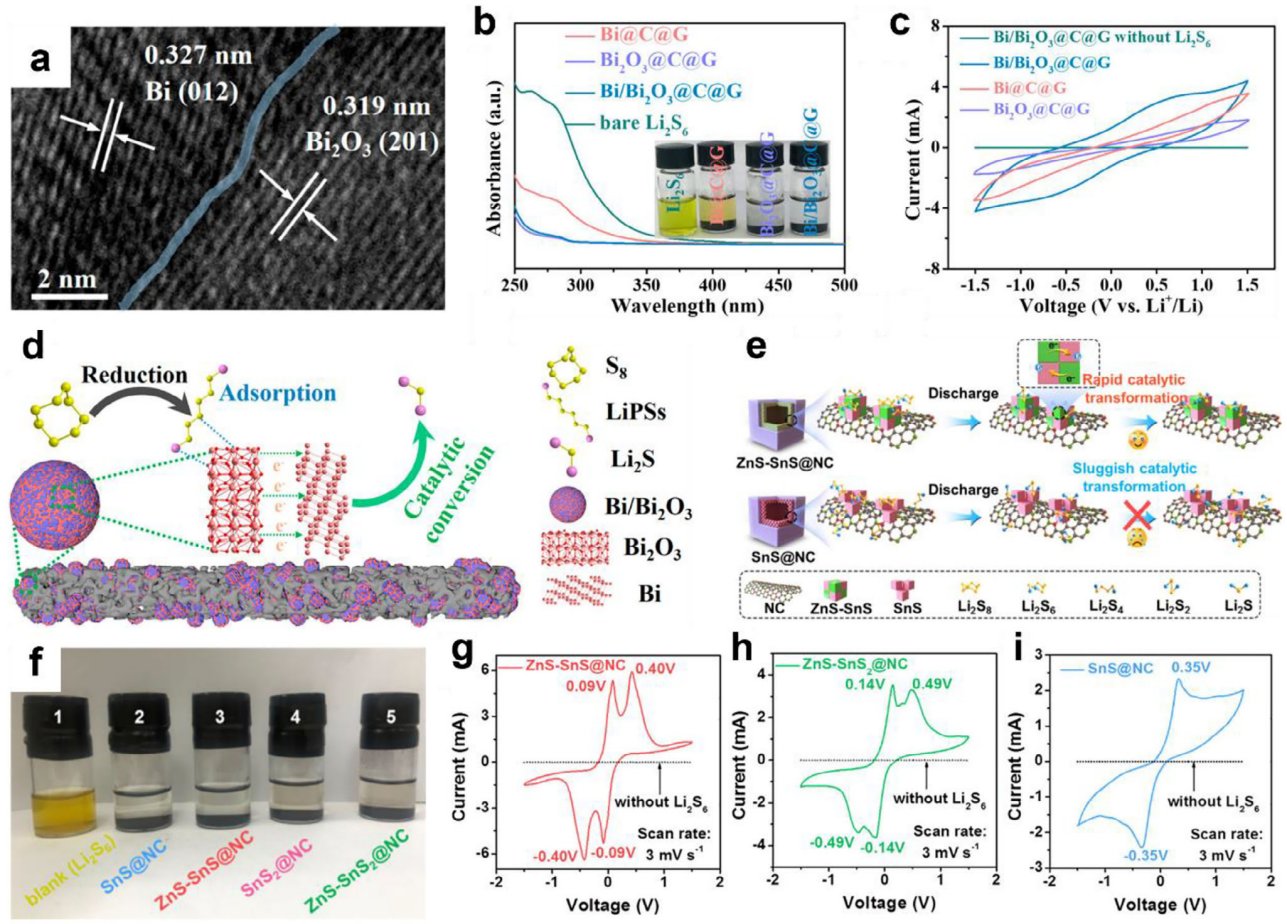
a) HRTEM images of Bi/Bi_2_O_3_@C@G. b) UV–vis spectra of Li_2_S_6_ solution before and after adsorption with Bi_2_O_3_@C@G, Bi/Bi_2_O_3_@C@G, and Bi@C@G. c) CV curves of symmetrical cells with electrodes coated with Bi_2_O_3_@C@G, Bi/Bi_2_O_3_@C@G, and Bi@C@G with and without Li_2_S_6_. d) Schematic illustrations of sulfur species transformation process on Bi/Bi_2_O_3_@C@G. (a–d) Reproduced with permission.^[^ ^93^ ^]^ Copyright 2021, American Chemical Society. e) Schematic illustrations of sulfur species transformation process on ZnS‐SnS@NC and SnS@NC. f) Digital photographs of LiPSs adsorption experiment. CV plots of Li_2_S_6_ symmetric cells with g) ZnS‐SnS@NC, h) ZnS‐SnS_2_@NC, and i) SnS@NC. (e–i) Reproduced with permission.^[^ ^94^ ^]^ Copyright 2021, American Chemical Society.

Heterostructure construction has been widely investigated for application in the cathode system of LSBs. Huang's group prepared a hierarchical sulfur host (Bi/Bi_2_O_3_@C@G) with a Bi/Bi_2_O_3_ heterogeneous structure by calcining the Bi‐MOF/graphene composite.^[^ ^93^ ^]^ Bi_2_O_3_ formed strong chemical adsorption with LiPSs through Bi‐S and O‐Li bonds, effectively anchoring dissolved LiPSs and suppressing the shuttle effect (**Figure** **10**a). Meanwhile, Bi had high conductivity and abundant catalytic sites, which enhanced Li‐ion diffusion and accelerated the redox kinetics of LiPSs (Figure 10b,c). Thus, Bi/Bi_2_O_3_ heterointerface combined the strong adsorption capability of Bi_2_O_3_ and the high catalytic activity of Bi well (Figure 10d), greatly improving the electrochemical performance of LSBs. The Bi/Bi_2_O_3_@C@G‐S with sulfur loading of 1.6 mg cm^−2^ delivered a high initial capacity of 1226.6 mAh g^−1^ at 0.2 C, retaining 1165.5 mAh g^−1^ capacity after 200 cycles.

In addition to sulfur hosts, heterostructure also demonstrates great potential in the design of interlayers between the cathode and the separator. Yao et al. developed a ZnS‐SnS@NC heterostructure coated with N‐doped carbon (ZnS‐SnS@NC) via a hydrothermal and sulfurization process and applied it as a bifunctional interlayer for LSBs.^[^ ^94^ ^]^ The ZnS‐SnS coupled heterointerfaces possessed a synergistic effect of lithiophilicity‐sulfiphilicity, which not only enabled the chemical adsorption and catalytic conversion of LiPSs but also promoted the rapid diffusion and uniform deposition of Li‐ion (Figure 10e). Specifically, SnS effectively anchored LiPSs through Sn‐S bonding (Figure 10f) while its intrinsic conductivity facilitated Li‐ion migration. Meanwhile, ZnS enhanced the catalytic activity and accelerated the redox kinetics of LiPSs (Figure 10g–i).

#### 6.1.2 Multicomponent Incorporation

**Figure 11:**
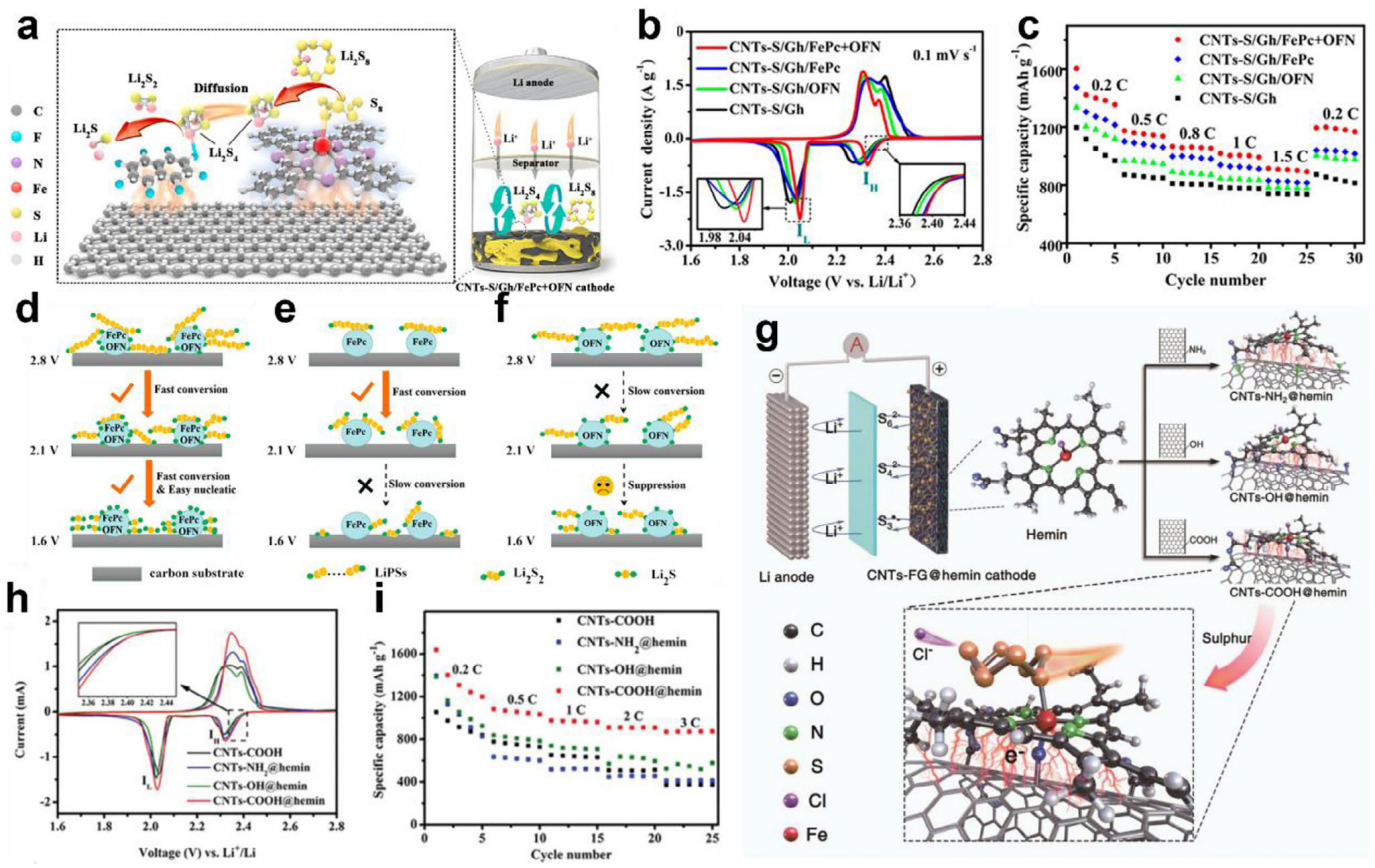
a) Schematic configuration of LSBs based on the Gh/FePc+OFN cathode. b) The fourth cycle of CV profiles and c) rate capabilities for CNTs‐S/Gh, CNTs‐S/Gh/OFN, CNTs‐S/Gh/FePc, and CNTs‐S/Gh/FePc+OFN cathodes. The schematic diagrams to show the adsorption and conversion mechanisms of LiPSs at the surface of d) CNTs‐S/Gh/FePc+OFN, e) CNTs‐S/Gh/FePc, and f) CNTs‐S/Gh/OFN, respectively. (a–f) Reproduced with permission.^[^ ^95^ ^]^ Copyright 2020, American Chemical Society. g) Schematic configuration of a LSBs based on three CNTs‐FG@hemin cathodes (FG═NH_2_, OH, COOH), and the mechanism of polysulfide adsorption at the CNTs‐COOH@hemin cathode. h) The second cycle of CV profiles and i) rate capacity of CNTs‐COOH, CNTs‐NH_2_@hemin, CNTs‐OH@hemin, and CNTs‐COOH@hemin cathodes. (g–i) Reproduced with permission.^[^ ^96^ ^]^ Copyright 2020, Wiley‐VCH.

In contrast to heterostructure construction, multicomponent incorporation combines multiple functional materials by physical or chemical methods to build composite structures, achieving a synergistic effect between lithiophilicity and sulfiphilicity. Nowadays, the typical form of multicomponent incorporation is catalyst@polar materials, which play a crucial role in constructing lithiophilic‐sulfiphilic microenvironments. The catalyst provides abundant active sites to accelerate the conversion of LiPSs while the polar matrix precisely anchors LiPSs through chemical adsorption and promotes Li‐ion transport. Huang's group constructed a ternary composite (Gh/FePc+OFN) by sequentially decorating graphene (Gh) with iron phthalocyanine (FePc) and octafluoronaphthalene (OFN) via ultrasonication.^[^ ^95^ ^]^ Gh/FePc+OFN, with the synergistic advantage of FePc and OFN, significantly inhibited the shuttle effect of LiPSs and accelerated the sulfur reaction kinetics (**Figure** **11**a). On the one hand, the sulfiphilic FePc chemically anchored sulfur species through Fe‐S coordination and catalyzed the conversion of long‐chain LiPSs (Li_2_S_8_, Li_2_S_6_) (Figure 11b). On the other hand, OFN enhanced lithiophilicity via Li‐F bonding, effectively immobilizing lithium species and promoting the nucleation and growth of short‐chain LiPSs (Li_2_S_4_, Li_2_S_2_ Li_2_S) to Li_2_S (Figure 11c–f). As a result, the CNTs‐S/Gh/FePc+OFN cathode delivered a high initial discharge capacity of 962 mAh g^−1^ at 1 C and retained 423 mAh g^−1^ with a low‐capacity decay of 0.055% per cycle after 1000 cycles. Huang's group developed a highly effective biomimetic mediator for LSBs by incorporating hemin bioenzyme onto CNTs‐COOH.^[^ ^96^ ^]^ The CNTs‐COOH@hemin exhibits excellent adsorption of LiPSs through the coordination of Fe(III) in a Fe‐O bond, enhancing the conversion rate of long‐chain LiPSs into Li_2_S. (Figure 11g,h). When utilizing this novel material, a remarkable initial specific capacity of 1637.8 mAh g^−1^ at 0.2 C, as well as outstanding long‐term cycling stability, with a minimal fading rate of only 0.042% per cycle over 1800 cycles (Figure 11i).

#### 6.1.3 Vacancy

Vacancy are one of the most fundamental structure regulation, which can modulate the local electronic structure by removing partial atoms or ions, thus enhancing the dual affinity of bulk materials. Among them, anion vacancy, such as oxygen vacancies and sulfur vacancies, have been widely applied in the modification of LSBs. Men et al. developed carbon nanofibers/CoS_2‐x_ compounds enriched with sulfur vacancies (CNFs/CoS_2‐x_) through steps of electrospinning, carbonization, sulfurization, and hydrogen reduction and coated them on separators for LSBs.^[^ ^97^ ^]^ The abundant sulfur vacancies induced the partial oxidation of Co^2+^ to Co^3+^, resulting in charge imbalance. This not only enhanced the chemical adsorption capacity for LiPSs but also reduced the nucleation barrier for Li_2_S deposition, thus effectively inhibiting the shuttle effect and accelerating sulfur reaction kinetics. In addition, sulfur vacancies created electron‐rich regions around the Co sites, which lowered the charge transfer resistance and improved the transport behavior of Li‐ion. As a result, the LSBs with the CNFs/CoS_2‐x_ modified separators exhibited outstanding rate performance and cycling stability, attaining a high capacity of 528 mAh g^−1^ even after 370 cycles at 1 C.

**Figure 12:**
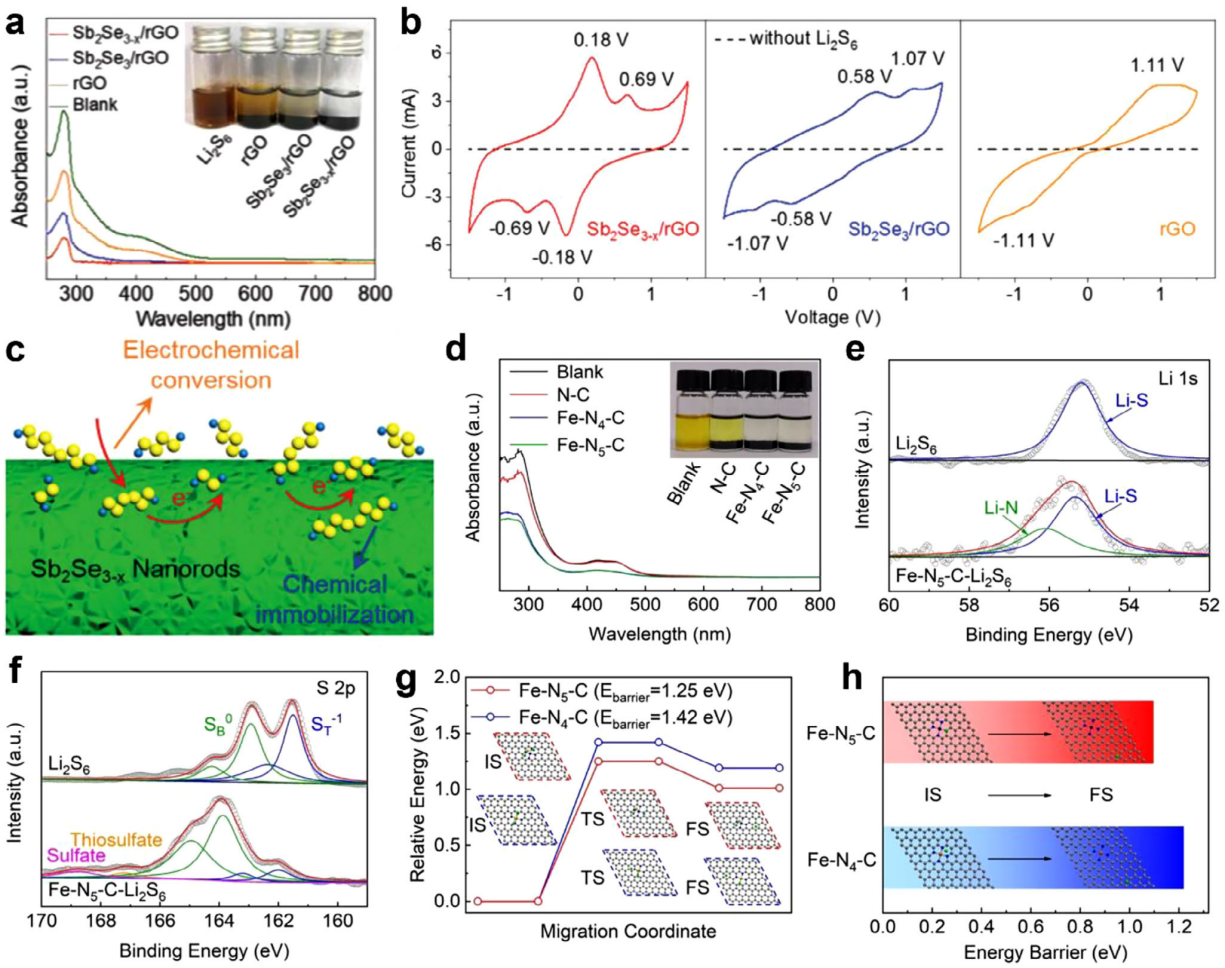
a) Optical observation of LiPS adsorption by different catalysts and the corresponding UV–vis spectra of the supernatants. b) CV curves of symmetric cells with different electrodes. c) Schematic illustration of the LiPSs conversion on the Sb_2_Se_3‐x_ surface. (a–c) Reproduced with permission.^[^ ^98^ ^]^ Copyright 2019, Wiley‐VCH. d) UV–vis spectra of Li_2_S_6_ solution soaked by N‐C, Fe‐N_4_‐C, and Fe‐N_5_‐C. High‐resolution XPS spectra of e) Li 1s and f) S 2p of Li_2_S_6_ and Fe‐N_5_‐C separated from the visualized adsorption test. Energy profiles of g) Li_2_S decomposition and h) Li‐ion migration on the surface of Fe‐N_4_‐C and Fe‐N_5_‐C. (d–h) Reproduced with permission.^[^ ^104^ ^]^ Copyright 2021, Wiley‐VCH.

In addition to anion vacancies, cation vacancies, represented by metal elements, have also demonstrated great potential in constructing lithiophilic‐sulfiphilic microenvironments. Tian et al. prepared a selenium‐deficient antimony selenide (Sb_2_Se_3‐x_)/reduced graphene oxide (rGO) composite (Sb_2_Se_3‐x_/rGO) by spray‐drying method combined with chemical reduction and thermal shock treatment (**Figure** **12**a).^[^ ^98^ ^]^ The introduced Se vacancies (V_Se_) enhanced the lithiophilicity and sulfiphilicity of Sb_2_Se_3‐x_, which effectively limited LiPSs shuttling and accelerated sulfur redox kinetics (Figure 12b,c). Specifically, V_Se_ strengthened the chemical adsorption of LiPSs by shortening the S‐Sb and Li‐Se bonds. Simultaneously, the defective sites reduced the energy barrier for Li_2_S decomposition (0.46 vs 0.91 eV), promoting the catalytic conversion of LiPSs. Benefiting from these properties, the LSBs with the Sb_2_Se_3‐x_/rGO modified separator displayed a high initial discharge capacity of 1387 mAh g^−1^ at 0.1 C and maintained 847 mAh g^−1^ with a low‐capacity decay of 0.027% per cycle after 500 cycles at 1 C.

#### 6.1.4 Doping

Doping is another important strategy for establishing structure regulation. By introducing heteroatoms or ions, they can alter the electronic distribution and chemical activity of materials, thereby improving the conductivity and catalytic properties of raw materials. Depending on the type of dopants, doping is mainly categorized into anion doping (such as N, S, O) and cation doping (such as various metals), both of which have been widely explored to optimize the performance of LSBs. For example, Liu et al. synthesized nitrogen‐doped Co_9_S_8_ (N‐Co_9_S_8_) nanoparticles via a hydrothermal method and used them in the cathode of LSBs.^[^ ^99^ ^]^ N‐doping enhanced the adsorption capability of Co_9_S_8_ toward LiPSs, which was attributed to the tendency of lithium species in LiPSs to incorporate with N atoms, forming more stable Li‐N bonds and thereby effectively suppressing the shuttle effect. More importantly, N‐doping optimized the electronic structure of Co_9_S_8_, which improved its catalytic activity, lowered the Li_2_S decomposition barrier, and accelerated sulfur redox kinetics. Therefore, the N‐Co_9_S_8_/Li_2_S_6_ cathode with a high sulfur loading of 2 mg cm^−2^ exhibited excellent cycling performance, attaining a high capacity of 605 mAh g^−1^ after 1000 cycles at 1 A g^−1^.

Recently, cation doping has also attracted considerable attention. Ren et al. prepared Sn‐doped Fe_2_O_3_ nanospheres (Sn@Fe_2_O_3_) via a hydrothermal treatment with an optimized Sn: Fe molar ratio of 1:3.^[^ ^100^ ^]^ Sn‐doping expanded the lattice spacing of Fe_2_O_3_, facilitating high sulfur loading and rapid Li‐ion migration. Meanwhile, Sn‐doping introduced numerous defects and active sites, which not only enhanced the chemical anchoring of LiPSs but also accelerated the conversion kinetics of LiPSs through strong catalytic activity. Due to these doping advantages, the Sn@Fe_2_O_3_ 1:3 cathode delivered a high initial discharge capacity of 1190 mAh g^−1^ at 0.2 C and maintained 847 mAh g^−1^ with a capacity after 80 cycles at 0.5 C.

#### 6.1.5 Coordination Engineering

In recent years, SACs have been regarded as promising modified materials to realize lithophilicity‐sulfiphilicity engineering due to their high atomic utilization, excellent structural stability, and well‐defined catalytic centers.^[^ ^101^, ^102^ ^]^ However, most SACs have saturated coordination structures, which hinders electron exchange as well as catalytic conversion of LiPSs. Therefore, many researchers have introduced defect engineering to construct SACs with unsaturated coordination, enhancing their catalytic activity and adsorption capacity for LiPSs. Guo et al. designed a deficient‐coordinated single‐atom indium catalyst (SAIn@CNT) by liquid‐phase self‐assembly and subsequent high‐temperature pyrolysis process.^[^ ^103^ ^]^ The unsaturated In‐N_3_ coordination centers enhanced the adsorption capability of LiPSs through strong hybridization between In 4d and S 3p orbitals, effectively suppressing the shuttle effect. Compared to In‐N_4_, the d band center of In‐N_3_ shifted upward (−5.256 vs −5.427 eV), which reduced the activation energy for LiPSs conversion and the energy barrier for Li_2_S decomposition, thereby accelerating sulfur redox kinetics. Furthermore, introducing defects also endowed the N and In sites with electron‐rich properties, which lowered the interfacial resistance, facilitated the transport of Li‐ion, and optimized the lithophilicity of SAIn. Benefiting from this unsaturated coordination, the LSBs with the SAIn@CNT modified separator showed high specific energy, excellent rate performance, and outstanding cycling stability.

Apart from unsaturated coordination, oversaturated coordinated SACs can also regulate their dual‐affinity properties. Zhang et al. prepared an oversaturated Fe‐N_5_ single atom catalyst (Fe‐N_5_‐C) via an absorption‐pyrolysis method and applied it as a sulfur host for LSBs.^[^ ^104^ ^]^ Fe‐N_5_ formed strong chemisorption with LiPSs through Fe‐S and Li─N bonds, limiting the shuttle effect (Figure 12d). Simultaneously, the coordination structure of Fe‐N_5_ introduced abundant defects and active sites, which reduced the Li_2_S nucleation/decomposition energy barriers (Figure 12e,f) and accelerated the bidirectional conversion of LiPSs. In addition, the oversaturated coordination of N lowered the interfacial charge transfer resistance, promoting the rapid migration of Li‐ion (Figure 12g,h).

### 6.2 MOFs Catalysts Structure Regulation

MOFs hold significant potential for constructing lithiophilic‐sulfiphilic microenvironments in LSBs due to their abundant active sites and well‐ordered porous structures. However, the saturated coordination between metal centers and organic ligands in MOFs limits the accessibility of these active sites, hindering the chemical adsorption and catalytic conversion of LiPSs. To address this challenge, researchers have pursued structural modifications of MOFs through three primary strategies: 1) defect engineering, 2) confinement effect, and 3) integrated design.

#### 6.2.1 Defect Engineering

**Figure 13:**
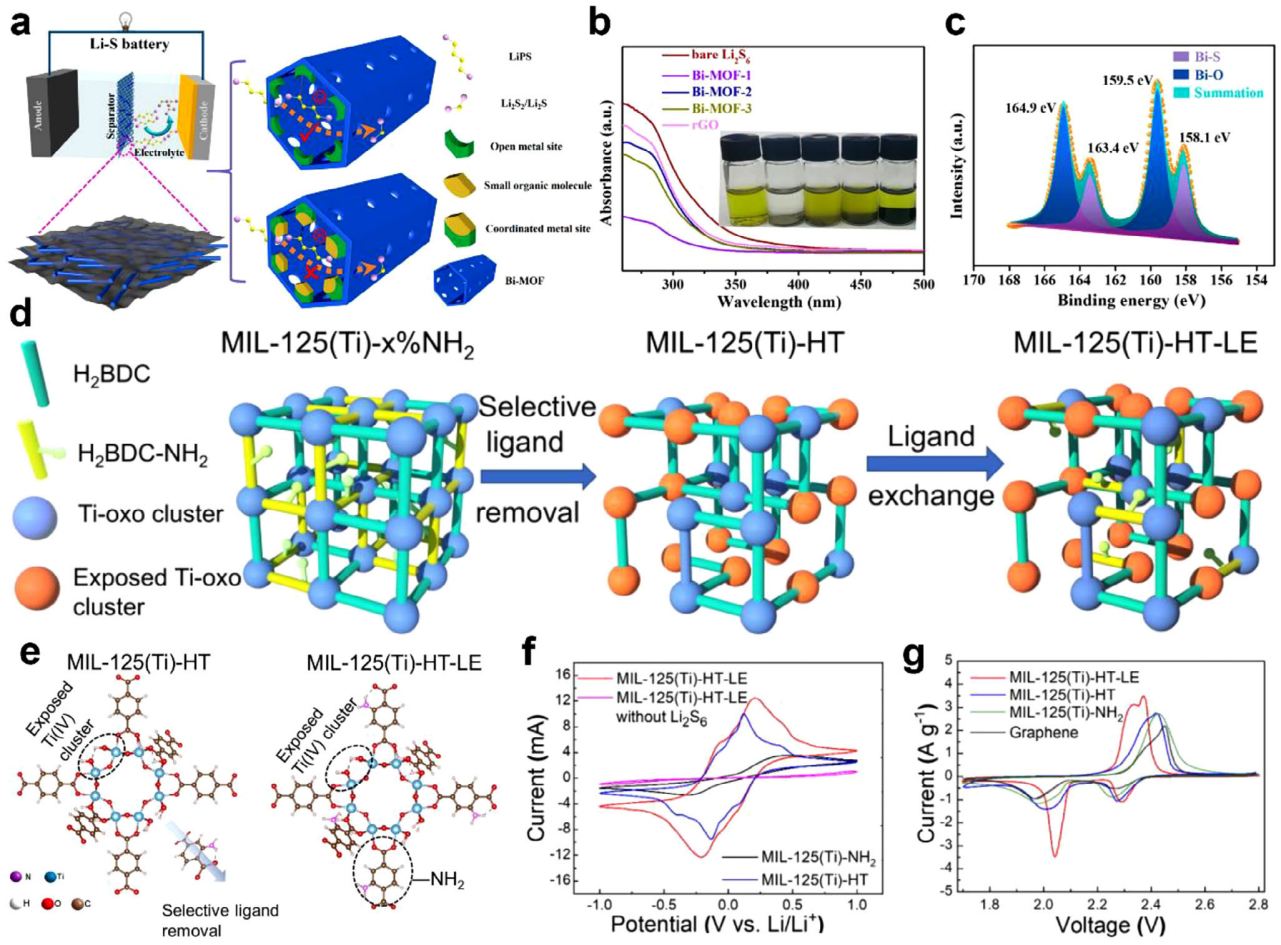
a) Regulation of active sites in Bi‐MOFs for LSBs. b) UV–vis spectra of Li_2_S_6_ solution before and after adsorption with rGO and different Bi‐MOFs. c) XPS spectrum of Bi 4f for Bi‐MOF‐1 adsorbed Li_2_S_6_. (a‐c) Reproduced with permission.^[^ ^105^ ^]^ Copyright 2021, American Chemical Society. d) The design strategy of Ti‐MTV‐MOF with both catalytic centers and adsorption sites. e) Structural illustration of Ti‐MTV‐MOFs. f) CV curves of symmetrical cells with different Ti‐MOFs. g) CV curves of LSBs with different Ti‐MOFs. (d‐g) Reproduced with permission.^[^ ^106^ ^]^ Copyright 2023, Elsevier.

Defect engineering has been introduced into MOFs to expose latent active sites by modifying their macrostructures or microstructures. Huang's group developed a Bi‐MOF‐1 with exposed open metal sites (OMSs) through coordination engineering remove H_2_O coordinated in metal center and applied it as a functional interlayer in LSBs.^[^ ^105^ ^]^ Bi^3+^ clusters in Bi‐MOF‐1 formed Bi‐S bonds with sulfur anions in LiPSs through Lewis acid‐base interactions (**Figure** **13**a), effectively adsorbing LiPSs (Figure 13b). Meanwhile, the exposed OMSs in Bi‐MOF‐1 significantly reduced the energy barrier of LiPSs conversion, facilitating the rapid nucleation/decomposition of Li_2_S (Figure 13c), thereby accelerating the redox kinetics. Therefore, the LSBs with the Bi‐MOF‐1/rGO@PP separator displayed outstanding electrochemical performance, delivering high initial capacities of 1050 and 1152 mAh g^−1^ at 0.1 C and 0.2 C, respectively.

Taking advantage of differences in the stability of coordinated ligands, Huang's group constructed a multifunctional MOF (MIL‐125(Ti)‐HT‐LE) with exposed catalytic metal clusters and amino adsorption ligands through selective ligand removal and subsequent ligand exchange (LE) (Figure 13d).^[^ ^106^ ^]^ The obtained MIL‐125(Ti)‐HT‐LE exhibited dual lithiophilic‐sulfiphilic properties, significantly enhancing the electrochemical performance of LSBs. Specifically, the exposed Ti‐oxo clusters chemically adsorbed sulfur species by d‐p orbital hybridization (Figure 13e) and provided open catalytic active sites to promote LiPSs conversion. Meanwhile, the introduced amino groups effectively anchored lithium species via Li‐N bonds, synergizing with the metal clusters to form an adsorption‐catalysis interface, thus suppressing the shuttle effect (Figure 13f) and accelerating redox reaction kinetics (Figure 13g). When evaluated as interlayers, the MIL‐125(Ti)‐HT‐LE displayed excellent rate performance and cycling stability, attaining a high capacity retention of 882.1 mAh g^−1^ even after 800 cycles at 1 C.

**Figure 14:**
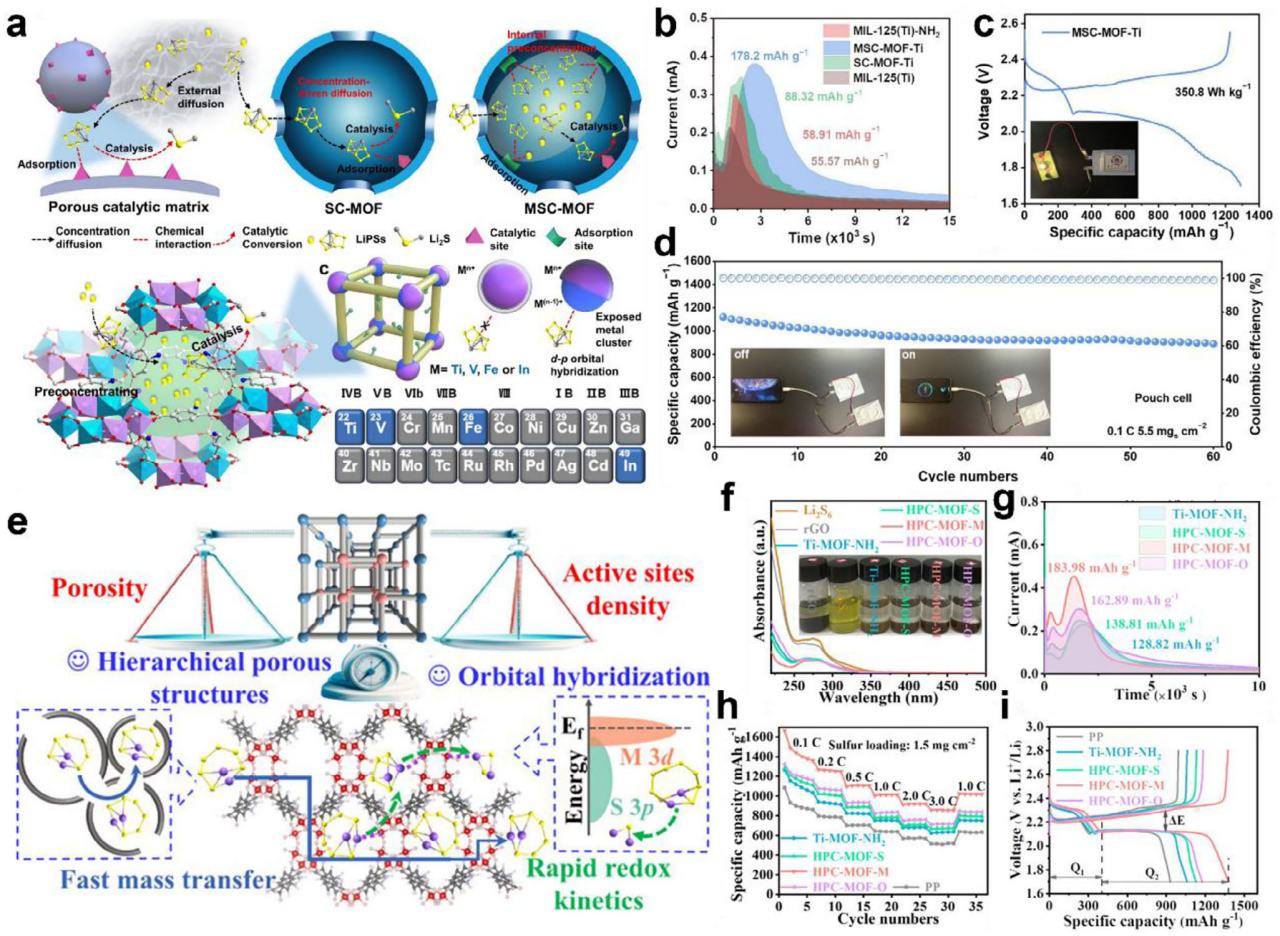
a) Schematic illustration of designed MSC‐MOF‐M for preconcentrating and catalyzing LiPSs. b) Potentiostatic discharge profiles of cells with electrodes coated with different Ti‐MOFs. c) Galvanostatic charge/discharge curves of high‐energy‐density pouch cell. d) Cycling performance of a single‐layer pouch cell at 0.1 C. (a–d) Reproduced with permission.^[^ ^107^ ^]^ Copyright 2024, Wiley‐VCH. e) Schematic illustration of HPC‐MOFs design strategy for transferring and catalyzing LiPSs. f) UV–vis absorbance spectra of Li_2_S_6_ solution with addition of different adsorbent. g) Potentiostatic discharge profiles at a constant voltage of 2.05 V on different material surfaces. The h) rate performance and i) discharge/charge curves of LSBs with different interlayers. (e–i) Reproduced with permission.^[^ ^108^ ^]^ Copyright 2024, American Chemical Society.

Furthermore, Huang's group introduced an innovative class of multi‐site catalytic MOFs (MSC‐MOFs‐M), incorporating variable mixed‐valence metal sites (M = Ti, Fe, V, or In), synthesized by heat‐treating pristine MOFs in a reducing atmosphere (**Figure** **14**a).^[^ ^107^ ^]^ The nanocages of these MSC‐MOFs feature lithiophilic ─NH_2_ and sulfiphilic mixed‐valence metal sites, which together create a collaborative adsorption and catalytic interface. The ─NH_2_ effectively capture LiPSs, concentrating reactants within the nanocages and increasing their likelihood of interacting with catalytic sites (Figure 14b). Simultaneously, the exposed mixed‐valence metal centers, working alongside adjacent ─NH_2_, coadsorb LiPSs during the SRR, enhancing the catalytic effect and accelerating the conversion of LiPSs through d‐p orbital hybridization. These features enable LSBs incorporating the MSC‐MOF‐Ti interlayer to achieve a high specific capacity of 875.8 mAh g^−1^ at 3 C, a notable areal capacity of 11.57 mAh cm^−2^ under high sulfur loading (9.32 mg cm^−2^), and a high‐energy pouch cell with a density of 350.8 Wh kg^−1^ (Figure 14c,d).

Simultaneously, Huang's group developed a hierarchical porous catalytic MOF (HPC‐MOF‐M) by treating Ti‐MOF‐NH_2_ with tannic acid using a time‐controlled etching method (15 min optimal).^[^ ^108^ ^]^ HPC‐MOF‐M possessed highly exposed Ti‐oxo clusters, which enhanced the adsorption capacity for LiPSs through dual‐site chemical interactions (Ti‐S and O‐Li bonds), effectively suppressing the shuttle effect (Figure 14e,f). Meanwhile, Ti‐oxo catalytic clusters weakened the S‐S bonds, reducing the energy barrier for LiPSs conversion (Figure 14g) and the charge transfer resistance, thereby significantly accelerating the redox reaction kinetics. Therefore, the LSBs with the HPC‐MOF‐M interlayer exhibited a high initial discharge capacity of 1186.3 mAh g^−1^ at 0.2 C and showed capacity retention of 785.1 mAh g^−1^ after over 500 cycles at 1 C (Figure 14h,i).

#### 6.2.2 Confinement Effect

**Figure 15:**
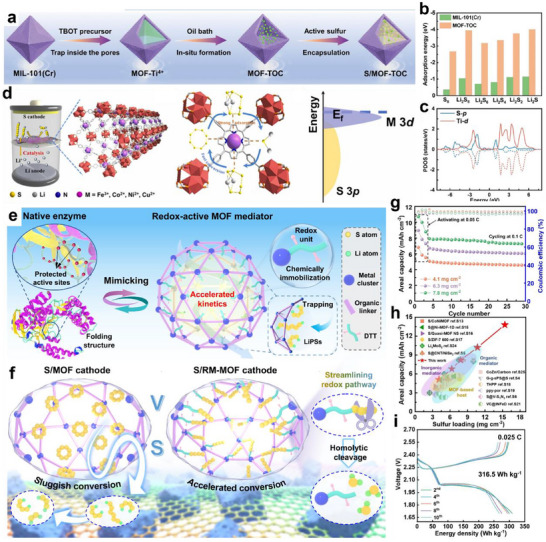
a) The preparation process of MOF‐TOCs. b) Adsorption energies of MOFs toward different sulfur species. c) PDOS of MOF‐TOC and S from Li_2_S_6_ after interaction. (a–c) Reproduced with permission.^[^ ^109^ ^]^ Copyright 2024, Wiley‐VCH. d) Regulation of single‐atom metal sites in PCN‐222(M)‐NS. (d) Reproduced with permission.^[^ ^110^ ^]^ Copyright 2023, Elsevier. Schematic illustration for e) the concept of the RM‐MOF and f) the different SRR pathways in the RM‐MOF and traditional MOF. g) Cycling stability of LSBs with S/rGO‐RM‐MOF. h) Areal capacities comparisons of coin‐cells with different cathodes. i) Charge/discharge profiles of the Ah‐level Li‐S pouch cell with S/rGO‐RM‐MOF cathode. (e–i) Reproduced with permission.^[^ ^111^ ^]^ Copyright 2025, Royal Society of Chemistry.

In addition to defect engineering, the loading of functional materials into MOFs structures via the confinement effect to construct lithiophilicity and sulfiphilicity MOFs catalysts has also received much attention. The confinement effect can be summarized in three perspectives: nanopores confinement, ligands confinement, and metal centers confinement. Huang's group constructed a novel MOF‐TOC composite by integrating sub‐nano Ti‐O clusters (TOCs) into the mesopores of MIL‐101 (Cr) and applied it as a sulfur host for LSBs.^[^ ^109^ ^]^ MOF‐TOC possessed great lithiophilic‐sulfiphilic bifunctional properties, significantly improving the electrochemical performance of LSBs (**Figure** **15**a). Specifically, TOCs chemically anchored sulfur and lithium species via Ti‐S and Li‐O bonds, respectively, which enhanced the adsorption capability of MOFs toward LiPSs and effectively confined the shuttle effect (Figure 15b). Additionally, TOCs facilitated Li‐ion migration and catalyzed the bidirectional conversion of LiPSs through d‐p orbital hybridization (Figure 15c). Due to the above merits, the S/MOF‐TOC cathode exhibited excellent rate performance and cycling stability, achieving a high discharge capacity of 805 mAh g^−1^ even after 500 cycles at 1 C.

To further enhance the activity and utilization of functional materials within MOFs, Huang's group developed a series of MOFs nanosheets (PCN‐222(M)‐NSs, M = Fe^3+^, Co^2+^, Ni^2+^, and Cu^2+^) by strategically incorporating SACS into the porphyrin ligand (Figure 15d).^[^ ^110^ ^]^ These nanosheets, featuring distinct M‐N_4_ single‐atom sites, were designed to modulate electronic interactions (d‐p orbital hybridization) with sulfur species, thereby optimizing the redox reaction kinetics and mitigating the shuttle effect of LiPSs in LSBs. Among these materials, PCN‐222(Cu)‐NS demonstrated the most pronounced ability to weaken S─S bond and accelerate SRR processes through effective d‐p orbital hybridization between the Cu‐N_4_ sites and LiPSs. These properties endowed LSBs incorporating PCN‐222(Cu)‐NS with high discharge capacities and exceptional long‐term cycling stability.

Additionally, Huang's group constructed a redox‐active metal‐organic framework mediator (RM‐MOFs) by chemically attaching the bilaterally active linear DTT to the exposed metal sites of MIL‐101(Cr). This framework serves as sulfur hosts and effective redox mediators for LSBs (Figure 15e,f).^[^ ^111^ ^]^ RM‐MOF effectively immobilizes LiPSs and directs their reduction reaction pathways via the redox‐active DTT unit, resulting in the formation of short‐chain organosulfur compounds and promoting subsequent radical reactions. This strategy significantly enhanced the performance of LSBs, increasing the discharge capacity by 150% compared to the unmodified LSBs. Furthermore, RM‐MOF ensures stable operation of pouch cells, achieving a high energy density of 316.5 Wh kg^−1^ (Figure 15g–i).

#### 6.2.3 Integrated Design

Lithiophilic‐sulfiphilic MOFs catalysts have demonstrated the ability to catalyze the conversion of LiPSs in LSBs. However, their insulating nature limits their overall catalytic efficiency. To address this, an integrated design of MOFs catalysts combining high electrical conductivity with lithiophilic‐sulfiphilic sites has been developed, enabling efficient and continuous catalysis for LiPS conversion.

**Figure 16:**
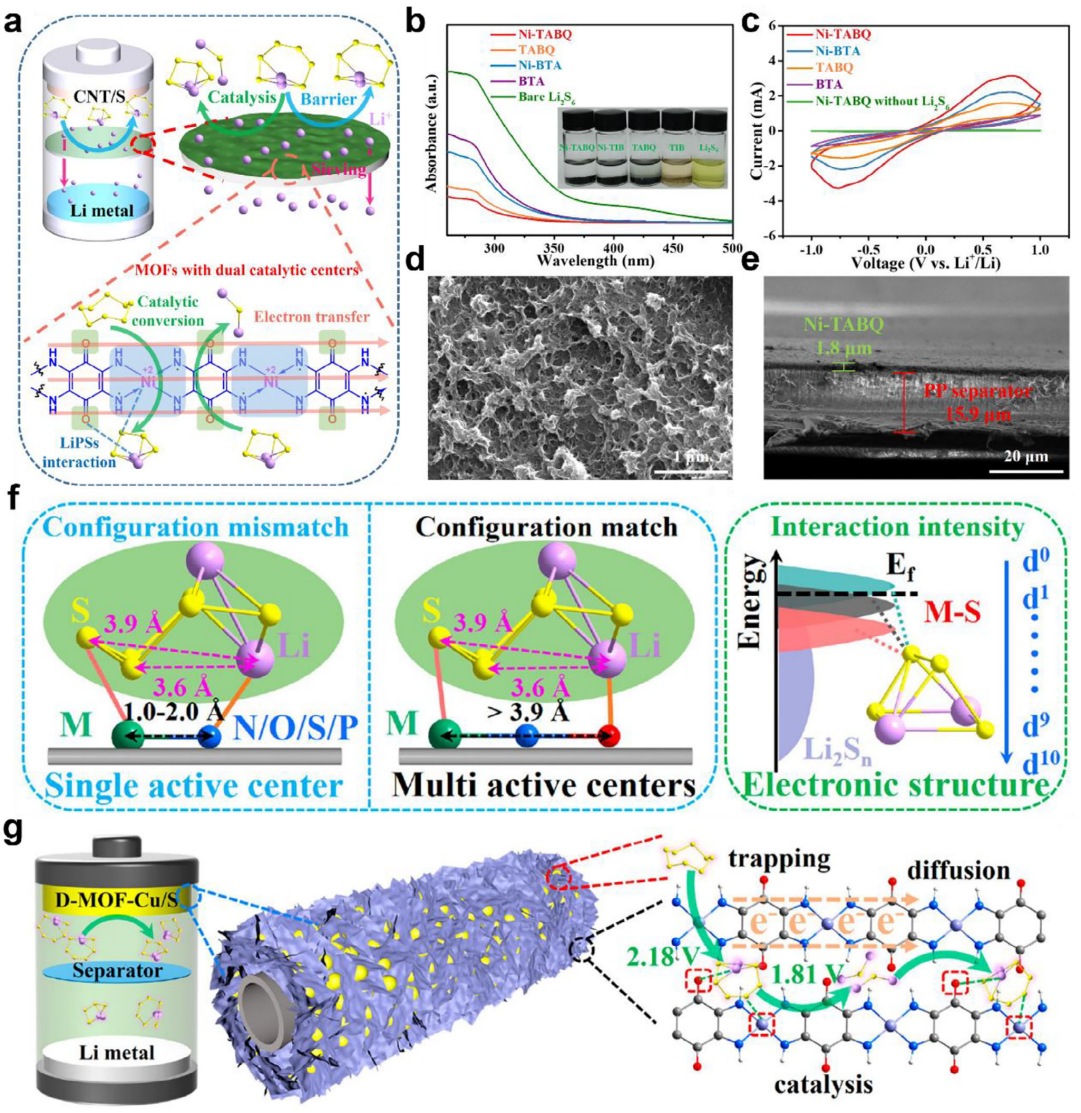
a) The design philosophy of catalytic MOF interlayer for the ions sieving and multistep catalytic conversion of LiPSs. b) UV–vis spectra of Li_2_S_6_ solution before and after adsorption. c) CV curves of symmetrical cells. d) SEM images of Ni‐TABQ interlayer on separator. e) Cross sectional SEM image of Ni‐TABQ membrane (0.076 mg cm^−2^). (a–e) Reproduced with permission.^[^ ^112^ ^]^ Copyright 2023, Elsevier. f) Illustration of configurational compatibility and electronic structure regulation between Li_2_S_6_ and different active centers. g) Schematic illustrations of sulfur species transformation processes on D‐MOF‐Cu. (f,g) Reproduced with permission.^[^ ^113^ ^]^ Copyright 2023, American Chemical Society.

Huang's group achieved a breakthrough by embedding lithiophilic‐sulfiphilic centers (Ni‐N_4_ and quinone groups) into a highly conductive MOF (Ni‐TABQ), and developed an ultrathin continuous Ni‐TABQ interlayer through in situ formation (**Figure** **16**a).^[^ ^112^ ^]^ This interlayer demonstrated strong adsorption sites, dual catalytic centers, superior electron transfer capabilities, and a compact microstructure (Figure 16b,c). These features allow it to effectively trap LiPSs, selectively sieve polysulfides, and accelerate redox reaction kinetics across various sulfur reaction steps (Figure 16d,e). As a result, LSBs equipped with the Ni‐TABQ interlayer exhibited significantly improved rate performance and exceptional long‐term cycling stability, with an ultralow capacity decay rate of 0.0198% over 1000 cycles at a 1 C rate.

Analogously, Huang's group introduced another innovative strategy by tailoring the configuration and electronic structure of conductive MOFs with lithiophilic‐sulfiphilic sites (D‐MOFs‐Cu), to develop an efficient catalytic sulfur host for LSBs (Figure 16f).^[^ ^113^ ^]^ The D‐MOFs‐Cu nanosheets, with their abundant lithiophilic‐sulfiphilic centers and electron transfer capabilities through π‐d conjugation, effectively store sulfur, trap LiPSs, and facilitate LiPSs conversion (Figure 16g). The multiscale design of D‐MOF‐Cu significantly enhanced LSBs performance, delivering improved Coulombic efficiency, stable cycling performance, and a high areal capacity of 10.04 mAh cm^−2^ under conditions of elevated sulfur loading and limited electrolytes, showcasing the effectiveness of these tailored lithiophilic‐sulfiphilic conductive MOFs.

## 7 Summary and Perspectives

This review presents a comprehensive analysis of various microenvironment engineering strategies for LSBs, categorizing them into four main approaches: 1) structural microenvironment engineering; 2) lithiophilicity microenvironment engineering; 3) sulfiphilicity microenvironment engineering, and 4) lithiophilicity‐sulfiphilicity microenvironment engineering. While these strategies offer significant potential in addressing the challenges of LSBs, a deeper understanding of their distinct characteristics and synergies is essential for further advancements in the field.

1. **Structural microenvironment engineering**: Traditionally, carbon‐based hosts have been favored for their high conductivity and ease of fabrication. However, issues like sulfur leaching and low energy density due to inactive host materials persist. MOFs and COFs, with their ordered porous structures, provide a distinct advantage in trapping LiPSs, but their poor conductivity hinders Li^+^ migration. Crucially, structural modifications alone are insufficient, as they lack the necessary chemical functionalities (lithiophilic/sulfiphilic sites) to enhance LiPSs management and reaction kinetics. Future efforts should focus on developing sulfur hosts that minimize non‐active content, such as porous conductive carbon frameworks combined with functional groups to enhance interactions with LiPSs and optimize both structural and electrochemical performance.
2. **Lithiophilicity microenvironment engineering**: The lithiophilicity strategy, which involves heteroatom doping, polar materials, and functional groups, improves Li^+^ affinity, promotes Li^+^ diffusion, and enhances electrolyte wetting. However, a critical challenge lies in the fact that lithiophilic strategies primarily interact with lithium species rather than sulfur species in LiPSs. This limits their ability to catalyze the conversion of LiPSs, which is central to mitigating the shuttle effect. Therefore, integrating lithiophilic modifications with sulfiphilic strategies could be a promising avenue for further exploration.
3. **Sulfiphilicity microenvironment engineering**: The sulfiphilicity approach enhances the interaction between sulfur and the host material through metal centers or organosulfur copolymers, improving the chemical adsorption and catalytic conversion of LiPSs. While this approach accelerates the redox kinetics of sulfur, their poor conductivity remains a significant limitation. Recent developments, such as carbon‐sulfur composites and hybrid materials with both sulfiphilic and conductive properties, aim to address this issue by enhancing both adsorption and conductivity. However, the complex interplay between sulfiphilicity and conductivity necessitates further investigation to identify the optimal balance.
4. **Lithiophilicity‐sulfiphilicity microenvironment engineering**: The combined approach of lithiophilic and sulfiphilic properties represents the most advanced strategy. It aims to optimize both Li^+^ transport and LiPSs adsorption, showing improved performance in suppressing the shuttle effect and enhancing reaction kinetics. However, the dynamic interaction and potential competition between lithiophilic and sulfiphilic sites during multi‐step reaction process remain poorly understood and difficult to characterize, posing a significant challenge for rational design and optimization.

While the reviewed microenvironmental engineering strategies have contributed to improving the electrochemical performance of LSBs, several core challenges still hinder their scalability and commercial viability. Understanding the root causes of these limitations is crucial for further progress. Below are some of the key challenges:

1. **Soluble long‐chain LiPSs conversion**: The slow conversion kinetics and shuttle effect of soluble long‐chain LiPSs remain significant obstacles in LSBs performance. While various strategies involving functional sites (lithiophilicity and sulfiphilicity microenvironment engineering) have optimized this process, they still fail to bypass the long‐chain LiPSs conversion altogether. Two potential solutions to overcome these limitations include: 1) Developing high‐performance catalysts that directly catalyze the conversion of sulfur species, skipping the intermediate soluble phase; and 2) Optimizing electrochemical pathways through techniques such as grafting sulfur species, utilizing small sulfur molecules, or enabling direct solid‐to‐solid conversion, thus bypassing the formation of soluble LiPSs.
2. **Reduced energy density due to inactive carbonaceous 3D platforms**: Carbonaceous 3D platforms, such as CNTs or graphene, are widely used to improve conductivity and provide a large surface area for sulfur deposition. However, one major drawback of these materials is their large volume/mass without contributing to the electrochemical reaction, limiting the overall energy density of the battery. Future studies should prioritize novel materials that can be used to minimize mass without sacrificing conductivity, such as lightweight conductive polymers or hybrid conductive networks. Optimizing the morphology of 3D hosts to improve sulfur utilization while maintaining a low overall mass, making them address the energy density challenge and more suitable for practical applications in high‐performance LSBs.
3. **Tradeoff between lithiophilicity and sulfiphilicity engineering**: Although both lithophilic and sulfiphilic strategies have shown promise in improving the stability and cycling performance of LSBs, the conceptual synergy between them has yet to be fully realized. In many cases, lithiophilicity and sulfiphilicity engineering may not provide a true synergy, but rather represent a tradeoff between improving the interaction of lithium and sulfur species. Lithiophilicity engineering favor Li^+^ adsorption but can destabilize Li_2_S deposition. Sulfiphilicity engineering strengthen LiPSs binding but may hinder Li^+^ diffusion. These two strategies can conflict in terms of their chemical nature. Thus, this tradeoff between lithiophilicity and sulfiphilicity needs further exploration, as the combined approach might lead to suboptimal performance if the interactions are not balanced. Future research should focus on understanding these competing factors and develop strategies to either enhance the performance of each independently or find ways to integrate both properties in a manner that avoids performance degradation.

## Conflict of Interest

The authors declare no conflict of interest.
